# Modulation of macrophage inflammatory function through selective inhibition of the epigenetic reader protein SP140

**DOI:** 10.1186/s12915-022-01380-6

**Published:** 2022-08-19

**Authors:** Mohammed Ghiboub, Jan Koster, Peter D. Craggs, Andrew Y. F. Li Yim, Anthony Shillings, Sue Hutchinson, Ryan P. Bingham, Kelly Gatfield, Ishtu L. Hageman, Gang Yao, Heather P. O’Keefe, Aaron Coffin, Amish Patel, Lisa A. Sloan, Darren J. Mitchell, Thomas G. Hayhow, Laurent Lunven, Robert J. Watson, Christopher E. Blunt, Lee A. Harrison, Gordon Bruton, Umesh Kumar, Natalie Hamer, John R. Spaull, Danny A. Zwijnenburg, Olaf Welting, Theodorus B. M. Hakvoort, Anje A. te Velde, Johan van Limbergen, Peter Henneman, Rab K. Prinjha, Menno P. J. de Winther, Nicola R. Harker, David F. Tough, Wouter J. de Jonge

**Affiliations:** 1 Tytgat Institute for Liver and Intestinal Research, Amsterdam Gastroenterology Endocrinology Metabolism Research Institute, Amsterdam University Medical Centers, University of Amsterdam, Amsterdam, the Netherlands; 2 Immuno-Epigenetics, Adaptive Immunity Research Unit, GlaxoSmithKline, Medicines Research Centre, Stevenage, UK; 3 Department of Pediatrics, Division of Pediatric Gastroenterology & Nutrition, Emma Children’s Hospital, Amsterdam University Medical Centers, University of Amsterdam, Amsterdam, the Netherlands; 4 Department of Oncogenomics, Amsterdam University Medical Centers, University of Amsterdam and Cancer Center Amsterdam, Amsterdam, the Netherlands; 5 Medicine Design, Medicinal Science and Technology, GlaxoSmithKline, Stevenage, UK; 6 Department of Clinical Genetics, Genome Diagnostics Laboratory, Amsterdam Reproduction & Development, Amsterdam University Medical Centers, University of Amsterdam, Amsterdam, the Netherlands; 7 GlaxoSmithKline, Cambridge, MA USA; 8 Constellation Pharmaceuticals, Cambridge, MA USA; 9 WuXi AppTec, Cambridge, MA USA; 10 Immunology Research Unit, GlaxoSmithKline, Medicines Research Centre, Stevenage, UK; 11 Department of Medical Biochemistry, Amsterdam University Medical Centers, University of Amsterdam, Amsterdam, the Netherlands; 12 Institute for Cardiovascular Prevention (IPEK), Munich, Germany; 13 Department of Surgery, University of Bonn, Bonn, Germany

**Keywords:** Macrophage, Crohn's disease, SP140

## Abstract

**Background:**

SP140 is a bromodomain-containing protein expressed predominantly in immune cells. Genetic polymorphisms and epigenetic modifications in the *SP140* locus have been linked to Crohn’s disease (CD), suggesting a role in inflammation.

**Results:**

We report the development of the first small molecule SP140 inhibitor (GSK761) and utilize this to elucidate SP140 function in macrophages. We show that SP140 is highly expressed in CD mucosal macrophages and in in vitro*-*generated inflammatory macrophages. SP140 inhibition through GSK761 reduced monocyte-to-inflammatory macrophage differentiation and lipopolysaccharide (LPS)-induced inflammatory activation, while inducing the generation of CD206^+^ regulatory macrophages that were shown to associate with a therapeutic response to anti-TNF in CD patients. SP140 preferentially occupies transcriptional start sites in inflammatory macrophages, with enrichment at gene loci encoding pro-inflammatory cytokines/chemokines and inflammatory pathways. GSK761 specifically reduces SP140 chromatin binding and thereby expression of SP140-regulated genes. GSK761 inhibits the expression of cytokines, including *TNF*, by CD14^+^ macrophages isolated from CD intestinal mucosa.

**Conclusions:**

This study identifies SP140 as a druggable epigenetic therapeutic target for CD.

**Supplementary Information:**

The online version contains supplementary material available at 10.1186/s12915-022-01380-6.

## Background

Although various therapeutic strategies provide benefit in Crohn’s disease (CD), the unmet need remains high with only approximately 30% of patients maintaining long-term remission [1–4]. Intestinal macrophages, which arise mainly from blood-derived monocytes, play a central role in maintaining gut homeostasis in health, but are also implicated as key contributors to CD [5, 6]. These opposing activities are linked to the capacity of macrophages to take on different functional properties in response to environmental stimuli. Epigenetic processes play an important role in the regulation of macrophage polarization/differentiation and gene expression, mediated by DNA methylation and histone post-translational modification [7, 8]. As such, epigenetic mechanisms mediated through the activity of specific histone acetylases and deacetylases activity are implicated in CD pathogenesis [9–11].

Speckled 140 kDa (SP140) is a nuclear body protein [12, 13] predominantly expressed in immune cells [14, 15]. Genome-wide association studies identified an association between single-nucleotide polymorphisms in *SP140* and CD [16] while rare *SP140*-associated variants have been identified in pediatric CD patients [17]. Further, we identified two differentially hyper-methylated positions at the *SP140* locus in blood cells of CD patients [18], suggesting aberrant regulation of its expression. Although little is known about how SP140 may be functionally linked to CD, a recent study reported that *SP140* knockdown in in vitro-generated inflammatory macrophages results a decrease in levels of pro-inflammatory cytokines [10]. Lower expression of *SP140* in intestinal biopsies was correlated with an improved response to anti-TNF therapy in CD patients [10]. Conversely, in biopsies from those who showed resistance to anti-TNF therapy, SP140 was highly expressed [10]. Together, these previous findings suggest a role for SP140 in CD pathogenesis.

SP140 protein harbors both a bromodomain (Brd) and a plant homeodomain (PHD) finger [10, 15] as illustrated in Fig. S1a. Since Brd and PHD domains are capable of binding to histones, this suggests that SP140 might function as an epigenetic reader. Readers are recruited to chromatin possessing specific epigenetic “marks” and regulate gene expression through mechanisms that modulate DNA accessibility to transcription factors (TFs) and the transcriptional machinery [19, 20]. Brds are tractable to small molecule antagonists, with selective inhibitors of several Brd-containing proteins (BCPs) reported [20, 21]. Such compounds have helped elucidate the function of their BCP targets; for instance, multiple inhibitors targeting BRD2, BRD3, and BRD4 have been used to demonstrate the role of these proteins in selectively regulating gene expression in the context of cancer and inflammation [9, 22, 23]. To date, no inhibitors targeting SP140 have been reported.

Here, we describe the discovery of the first specific SP140 inhibitor and utilize this compound to investigate the functional relevance of SP140 in CD pathogenesis, focusing on its role in macrophages. We show that SP140 functions as an epigenetic reader in controlling the generation of an inflammatory macrophage phenotype, and in regulating the expression of pro-inflammatory and CD-associated genes. We show that GSK761 inhibits SP140 binding to an array of inflammatory genes and demonstrate its efficacy in CD colon macrophages ex vivo, presenting evidence for SP140 as a target for regulating inflammation in CD. In addition, this study suggests that SP140 inhibition may also serve to support anti-TNF therapy in non-responder CD patients.

## Results

### Elevated SP140 expression in inflammatory diseases and mucosal macrophages of CD patients

**Fig. 1:**
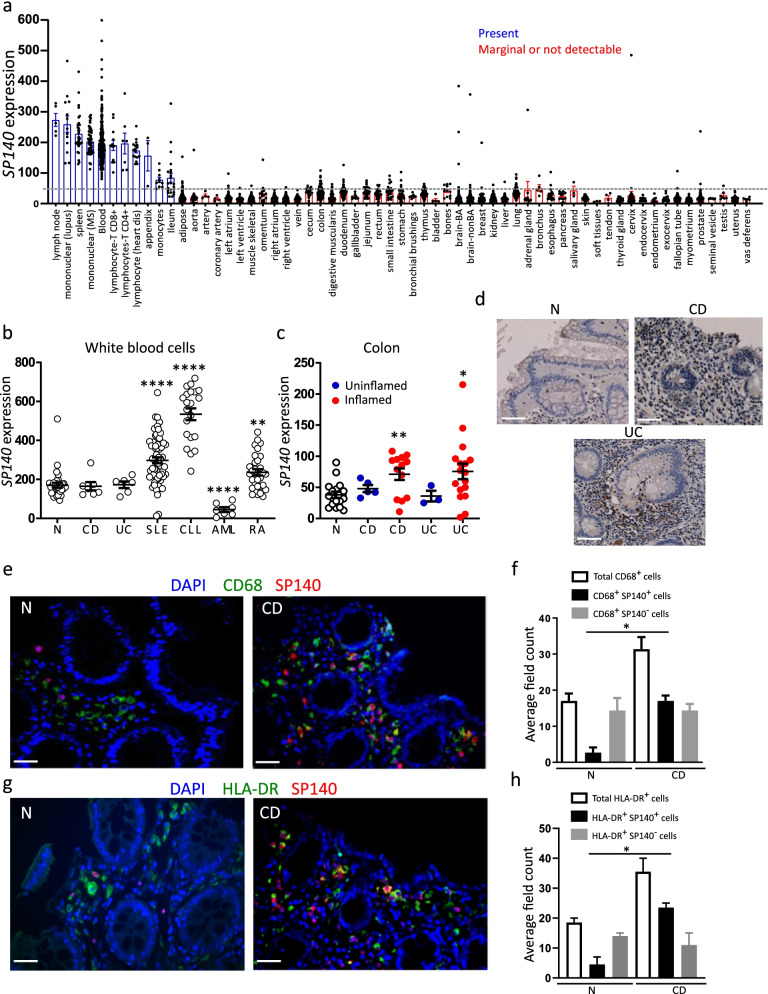
SP140 expression associates with inflammatory diseases and mucosal macrophages of CD patients. **a ***SP140* gene expression in human tissues and cell types as indicated. Expression is given as count normalized with MAS5.0. **b ***SP140* gene expression in white blood cells of normal healthy controls (N) (*n*=33), Crohn’s disease (CD) (*n*=6), ulcerative colitis (UC) (*n*=6), systemic lupus erythematosus (SLE) (*n*=64), chronic lymphocytic leukemia (CCL) (*n*=21), acute myeloid leukemia (AML) (*n*=7), and rheumatoid arthritis (RA) (*n*=32) patients. **c ***SP140* gene expression in human colon tissue obtained from N (*n*=18), non-inflamed and inflamed CD (*n*=5 and 13, respectively), and UC (*n*=3 and 17, respectively) colonic tissues. Data was collected from in-house GSK microarray profiler. Expression is given as count normalized with MAS5.0. **d** Immunohistochemistry of SP140 protein in colon tissue obtained from N and inflamed CD and inflamed UC tissue, scale bar: 100 μm. **e** Immunofluorescence staining of DAPI (blue), CD68 (green), and SP140 (red) in N or inflamed CD colon tissue, scale bar: 100 μm. **f** The average of mucosal cell count per 3 visual fields of total CD68^+^ macrophages, SP140^+^ CD68^+^ macrophages and SP140^−^ CD68^+^ macrophages in N and inflamed CD tissue (*n* = 3 patients per group). **g** Immunofluorescence staining of DAPI (blue), HLA-DR (green), and SP140 (red) in uninflamed or inflamed CD colon tissue, scale bar: 100 μm. **h** The average of mucosal cell count of 3 visual fields per tissue of total HLA-DR^+^ macrophages, SP140^+^ HLA-DR^+^ macrophages, and SP140^−^ HLA-DR^+^ macrophages in N and inflamed CD tissue (*n*=3 patients per group). Statistical significance is indicated as follows: *P < 0.05, **P < 0.01, ****P < 0.0001

*SP140* gene expression in a variety of cells and tissues was analyzed using an in-house GSK microarray profiler. *SP140* was predominantly expressed in blood, CD8^+^ and CD4^+^ T cells, monocytes, and secondary immune organs such as lymph node and spleen (Fig. 1a). We then investigated SP140 expression in white blood cells (WBC) and intestinal tissue of IBD patients versus controls. While *SP140* gene expression in WBC was comparable in IBD and healthy controls (Fig. 1b), SP140 gene and protein expression were found to be increased in inflamed colonic mucosa of both CD and UC patients (Fig. 1c, d). Notably, an increase in CD68^+^SP140^+^ and HLA-DR^+^SP140^+^ but not CD68^+^SP140^−^ or HLA-DR^+^SP140^−^ cells was observed in CD inflamed colonic mucosa compared to normal control mucosa or uninflamed tissue, respectively (Fig. 1e–h). Elevated SP140 expression was also found in other inflammatory conditions including in WBC of systemic lupus erythematosus and rheumatoid arthritis patients (Fig. 1b), and in inflamed tissues of appendicitis, sarcoidosis, psoriatic arthritis, rheumatoid arthritis, Hashimoto’s thyroiditis and Sjogren’s syndrome patients (Additional file 1: Fig. S1b). High expression of SP140 was also observed in chronic lymphocytic leukemia (Fig. 1b).

**Fig. 2:**
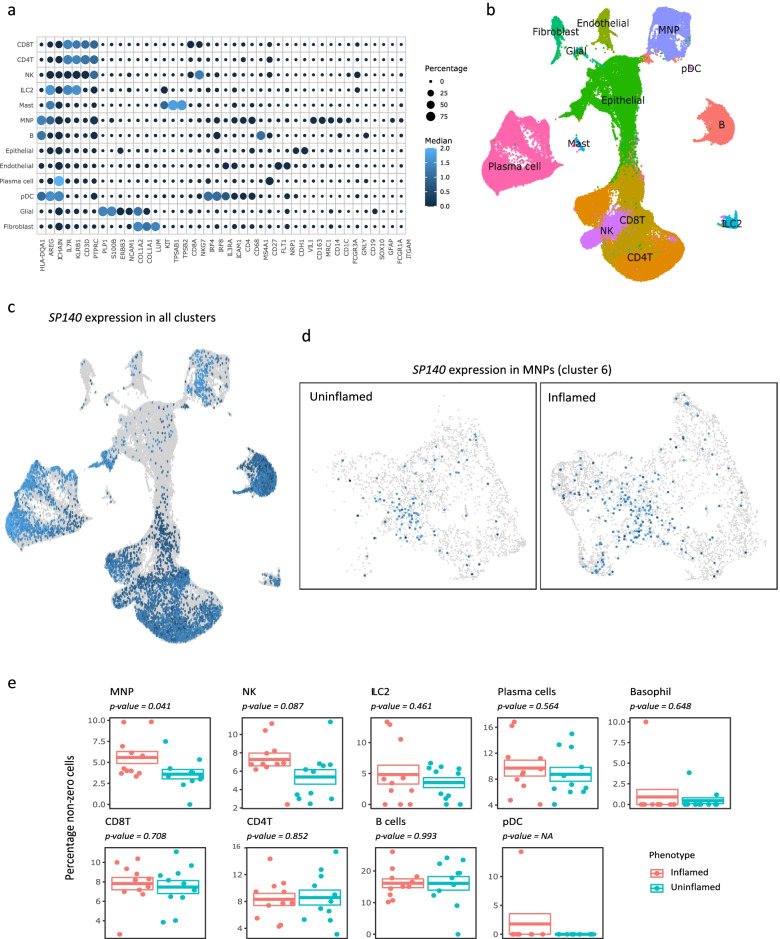
Single-cell analyses of *SP140* expression in inflamed and uninflamed CD ileum. Publicly available single-cell RNA sequencing [24] was used to illustrate *SP140* expression in intestinal (ileum) macrophages in inflamed (*n*=11) and uninflamed (*n*=11) tissue CD patients. **a** Marker genes expression per cell type where the size of the dots represents the percentage of cells expressing the gene. Darker blue represents more reads per cell. **b** UMAP of all the cells annotated with their annotated cell type. **c** Visual illustration of SP140 expression in all cells. Darker blue represents more reads per cell. **d** Visual illustration of the actual counts of SP140 expression in cluster 6 (MNPs). Darker blue represents more reads per cell. **e** Comparative analyses of the percentage cells expressing SP140 when comparing inflamed with uninflamed in different immune cells

Next, we analyzed a publicly available single-cell RNA sequencing experiment [24] of inflamed and uninflamed ileal tissue (biopsies) obtained from CD patients. Unsupervised clustering analysis identified 22 cluster blocks (Additional file 1: Figs. S2a and b). Based on different cell markers, we identified cell types existing in each cluster (Fig. 2a, b). Expectedly, *SP140* expression was predominantly observed in the immune cell compartment (Fig. 2c). Based on the expression of *CD14*, *CD16* (*FCGR3A)*, *CD1C*, *CD64 (FCGR1A)*, *CD68*, *CD163*, *CD206 (MRC1)*, *HLA-DR*, and *CLEC4A* (Fig. 2a and Additional file 1: Fig. S2c), we identified cluster 6 as containing monocytes, macrophages, and dendritic cells (Additional file 1: Fig. S2c and Fig. 2b), altogether representing mononuclear phagocytes (MNP). As per the observations made by Martin et al. [24], the abundance of the MNPs was significantly higher in inflamed ileal biopsies (Additional file 1: Fig. S2d), which accordingly manifested as an increased percentage of *SP140*-expressing MNPs in inflamed ileal biopsies (Fig. 2d, e). Interestingly SP140 expression did not differ significantly in other immune cells (including T and B cells) between inflamed and uninflamed tissues (Fig. 2e). Through subclassification analysis of the MNPs, we mapped the monocytes (*CD14* and *FCGR3A*) along their developmental trajectory towards macrophages (*CD68*, *CD163*, and *CD206*) or dendritic cells (*CD1C* and *CLEC4A*) (Additional file 1: Fig. S2e). Projecting the expression of *SP140* along the inferred trajectory suggests significant association with the developmental process (Additional file 1: Fig. S2f).

### SP140 mediates inflammatory “M1” macrophage function

**Fig. 3:**
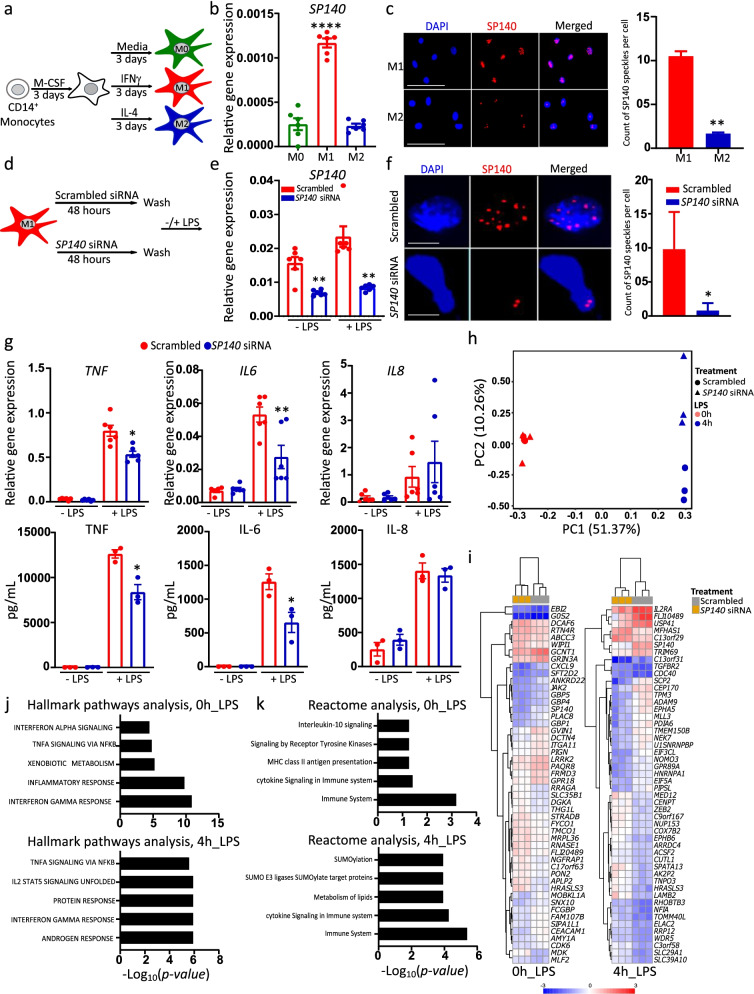
*SP140* knockdown reduces the activity of the inflammatory macrophages. **a** Scheme of polarization protocol of human CD14^+^ monocytes to “M0,” “M1,” and “M2” macrophage phenotypes, with indicated cytokines as described in the “Methods” section. **b** Relative gene expression of *SP140* in “M0,” “M1,” and “M2” macrophages, *n*=6. **c** Immunofluorescence staining of SP140 speckles in “M1” and “M2” macrophages imaged by microscopy (left) and quantified per nuclei as nuclear bodies count (right). Images were counted in 150 cells selected randomly from 3 different staining per condition (Total number of SP140 speckled in 150 cells/150 = average per cell), scale bar: 30 μm. **d** Scheme of *SP140* silencing protocol as described in “Methods” section. **e** The efficiency of *SP140* silencing was assessed by measuring relative gene expression (qPCR) of *SP140* (*n*=6) and **f** immunofluorescence staining of SP140 speckles nuclear bodies (left) and SP140 speckled nuclear bodies count (counted as described above) (right), scale bar: 3 μm. **g** Relative gene expression after LPS stimulation: (top, *n*=6), and LPS-induced protein levels (bottom, *n*=3) *n*=3, of TNF, IL-6, and IL-8. **h** PCA of gene expression dataset of LPS induced genes in scrambled- or *SP140* siRNA-treated “M1” macrophages (unstimulated or stimulated with 4 h 100 ng/mL LPS) assessed through microarray; PC1 represents most variance associated with the data (LPS stimulation) and PC2 represents second most variance (siRNA), *n*=3. **i** Heatmap of top 50 DEGs from microarray gene expression dataset of LPS induced genes in scrambled- or *SP140* siRNA-treated “M1” macrophages (unstimulated or stimulated with 4 h 100 ng/mL LPS) (non-annotated genes were not included). **j** Hallmark pathways analysis and **k** Reactome pathway analysis were carried out using ShinyGO v0.60 illustrating the most impacted pathways by *SP140* siRNA in unstimulated “M1” macrophages (bottom) or after 4 h of 100 ng/mL LPS stimulation (bottom). In all assays, statistical significance is indicated as follows: *P < 0.05, **P < 0.01, ***P < 0.001, ****P < 0.0001

Given the high SP140 expression in CD mucosal macrophages, we investigated whether SP140 is associated with inflammatory activation. Human CD14^+^ monocytes or THP-1 cells were differentiated into macrophages and then polarized into “M1” and “M2” phenotypes using IFN-γ and IL-4 respectively or left without treatment (M0) (Fig. 3a and Additional file 1: Fig. S3b). The polarization was verified by measuring gene expression of *CD64* and *CCL5* (markers of “M1” macrophages) and *CD206* and *CCL22* (markers of “M2” macrophages) (Additional file 1: Fig. S3a and b). *SP140* mRNA was expressed at significantly higher levels in “M1” compared to “M0” or “M2” macrophages derived from primary monocytes (Fig. 3b) or THP-1 cells (Additional file 1: Fig. S3c). Protein staining showed increased numbers of SP140 protein-containing speckles in the nucleus of “M1” compared to “M2” macrophages derived from both human monocytes (Fig. 3c) and THP-1 cells (Additional file 1: Fig. S3d). LPS induced an increase in *SP140* gene expression in “M0” and “M1” macrophages, along with enhanced *CCL5* and decreased *CCL22* gene expression (Additional file 1: Fig. S3e). The expression levels of 12 other BCPs (*SP140L*, *SP100*, *SP110*, *BRD2*, *BRD3*, *BRD4*, *BRD9*, *BAZ2A*, *BAZ2B*, *PCAF*, *EP300*, and *CREBBP*) showed no increase in “M1” compared to “M0” macrophages (Additional file 1: Fig. S4a), indicating that the association between *SP140* and inflammatory macrophages was not a common phenomenon among BCPs.

To investigate SP140 function, we initially used siRNA-mediated knockdown to reduce *SP140* expression in “M1” macrophages, achieving an approximately 75% reduction in mRNA (Fig. 3e) and a clear decrease in the number of SP140 protein-containing speckles (Fig. 3f). Knockdown seemed specific to *SP140*, as expression of related family members *SP110*, *SP100*, and *SP140L* was not affected (Additional file 1: Figs. S4b, b and c and Additional file 2: Table S1 and Additional file 3: Table S2). After the knockdown, cells were stimulated with LPS or kept unstimulated (Fig. 3d). *SP140* silencing led to a decrease in LPS-induced IL-6 and TNF mRNA and protein levels (Fig. 3g). After transcriptional profiling, principal component analysis (PCA) revealed that the variance was most associated with *SP140* siRNA treatment in LPS-stimulated “M1” macrophages (Fig. 3h). Downregulation of some key CD-associated genes, notably *CXCL9* [25], *CEACAM1* [26], and *JAK2* [27, 28], was apparent among *SP140*-siRNA-treated unstimulated macrophages, and *IL2RA* [29] and *TRIM69* [30] among *SP140*-siRNA LPS-stimulated macrophages (Fig. 3i). *SP140* silencing affected common inflammatory pathways, such as TNF signaling via NFKB, IFN-γ response, inflammatory response (Fig. 3j), and cytokine signaling (Fig. 3k).

### The development of a selective inhibitor of SP140 (GSK761)

**Fig. 4:**
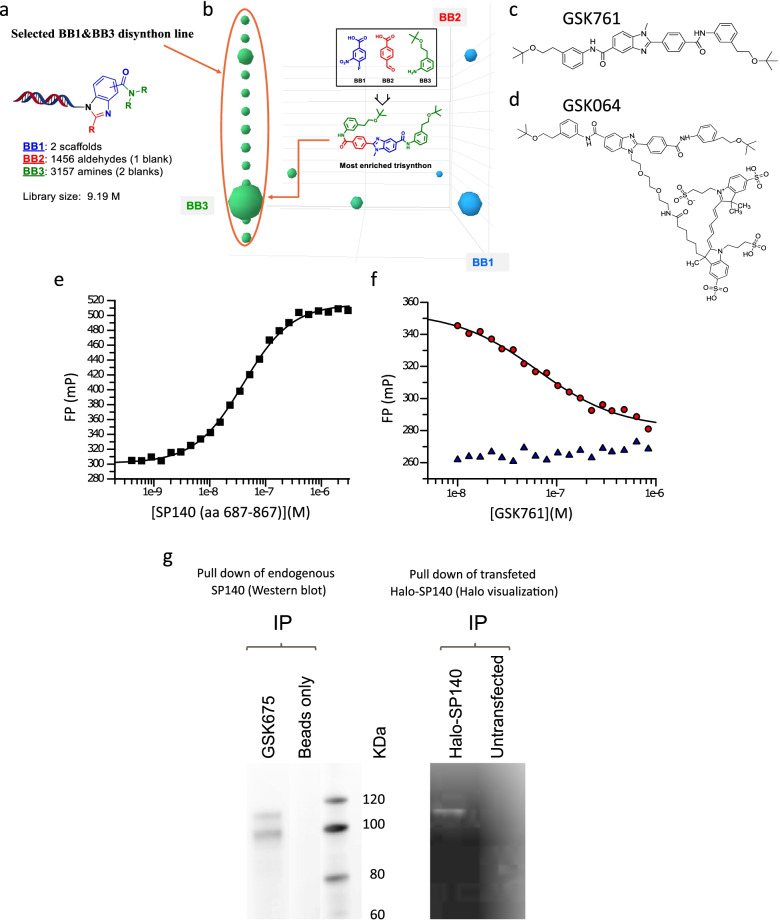
The Development of the first small molecule inhibitor of SP140 (GSK761) and investigating its affinity and selectivity. **a** Scheme of the three-cycle benzimidazole library and **b** Spotfire cube view of the SP140 selection output from the benzimidazole library. BB1, cycle 1 building blocks; BB2, cycle 2 building blocks; and BB3, cycle 3 building blocks. Each individual dot in the cube represents discrete small molecule warheads after 3 rounds of affinity selection, while the size of the dot corresponds to the number of unique instances recorded by DNA sequencing (2-24). The dots are colored by the BB3s that compose the library molecules. Library members with a single copy were removed to simplify visualization revealing a prominent line defined by a specific BB1&BB3 combination (disynthon). The most enriched trisynthon (BB1, BB2, and BB3 combination) represents the biggest dot on the line. **e** Biochemical characterization of the interaction between **c** GSK761 and recombinant SP140 in a fluorescence polarization (FP) binding assay using **d** a fluorophore-conjugated version of GSK761: GSK064 (generated by fluorescent labelling of GSK761). The mean binding affinity for this interaction was a *K*_d_ = 41.07 ± 1.42 nM (*n* = 5). **f** A FP-binding assay was configured using recombinant SP140 and GSK064, which was used to determine the potency of GSK761. Displacement of GSK064 from SP140 by GSK761 (circles) was achieved and determined to have a mean IC_50_ of 77.79 ± 8.27 nM (*n*=3). No effect on GSK064 motion was observed in the presence of varying concentrations of GSK761 (triangles)**.** The data presented in **d** and **e** are representative data from a single experimental replicate, affinities, and potencies are mean values determined from multiple test occasions. **g** Endogenous SP140 (HuT78 nuclear extracts) and Halo-tagged SP140 (transfected HEK29 cells) were pulled down using a biotinylated version of GSK761 (GSK675) and visualized by Western blotting and gel electrophoresis. Biotinylated beads only and SP140 untransfected cells were used as control

To identify selective SP140 binding compounds, we utilized encoded library technology to screen the GSK proprietary collection of DNA-linked small molecule libraries [31, 32]. Affinity selection utilizing a recombinant protein construct spanning the PHD and Brd domains of SP140 was carried out, leading to the identification of an enriched building block combination in DNA Encoded Library 68 (DEL68) (Fig. 4a). Representative compounds of the identified three-cycle benzimidazole chemical series were synthesized, which yielded the small molecule, GSK761 (Fig. 4b, c). More details describing GSK761 development are added in the “Methods” section. To quantify the interaction between GSK761 and SP140 (aa 687-867), the dissociation constant (*K*_d_) was determined using a fluorescence polarization (FP)-binding approach. A fluorophore-conjugated version of GSK761, GSK064 (Fig. 4d), was prepared and subsequently used to determine a *K*_d_ value of 41.07 ± 1.42 nM for the interaction with SP140 (aa 687-867) (Fig. 4e). To further validate this interaction, competition studies were carried out using GSK761 in a FP-binding assay configured using GSK064 and SP140 (aa 687-867). Competitive displacement of GSK064 from SP140 (aa 687-867) by GSK761 was observed, subsequently leading to the determination of an IC_50_ value of 77.79 ± 8.27 nM (Fig. 4f). Prior to utilizing GSK761 for cell-based binding studies to endogenous SP140, the cell penetration capacity of the compound was assessed using mass spectrometry and the methodology described by [33]. Concentration measurements showed that GSK761 had a pΔC_total_ value of 1.45 ± 0.21, indicating that GSK761 permeates human cells and accumulates intracellularly by an order of magnitude when compared to GSK761 free in solution.

To confirm the binding of GSK761 to full-length SP140, immobilized GSK761 was used to probe for SP140 in nuclear extracts from HuT78 cells and HEK293 cells transfected with Halo-tagged SP140. Biotinylated beads only and untransfected HEK293 cells were utilized as controls. Both endogenous and Halo-tagged SP140 were pulled down with biotinylated GSK761 and visualized via Western Blotting (Fig. 4g). Endogenous SP140 is observed as a doublet containing the four largest isoforms (predicted 86–96 kDa) [10] while Halo-tagged SP140 exhibits a single band. No bands were detected with beads only or in untransfected HEK293 cells. The specificity of GSK761 for SP140 was profiled using the BROMOscan® assay, in which DNA-tagged BCPs were incubated with an increasing concentration of GSK761 or DMSO. Binding is assessed by measuring the amount of bromodomain captured in GSK761 vs DMSO samples by using an ultra-sensitive qPCR. There was evidence of low affinity interaction between GSK761 and several tested BCPs, with binding detected at concentrations >21,000 nM (Additional file 4: Table S4). However, no binding was detected at concentrations ≤ 21,000 nM indicating a high degree of specificity of GSK761 for SP140 (Additional file 4: Table S4).

### GSK761 reduces the inflammatory activation of macrophages and expression of pro-inflammatory cytokines

**Fig. 5:**
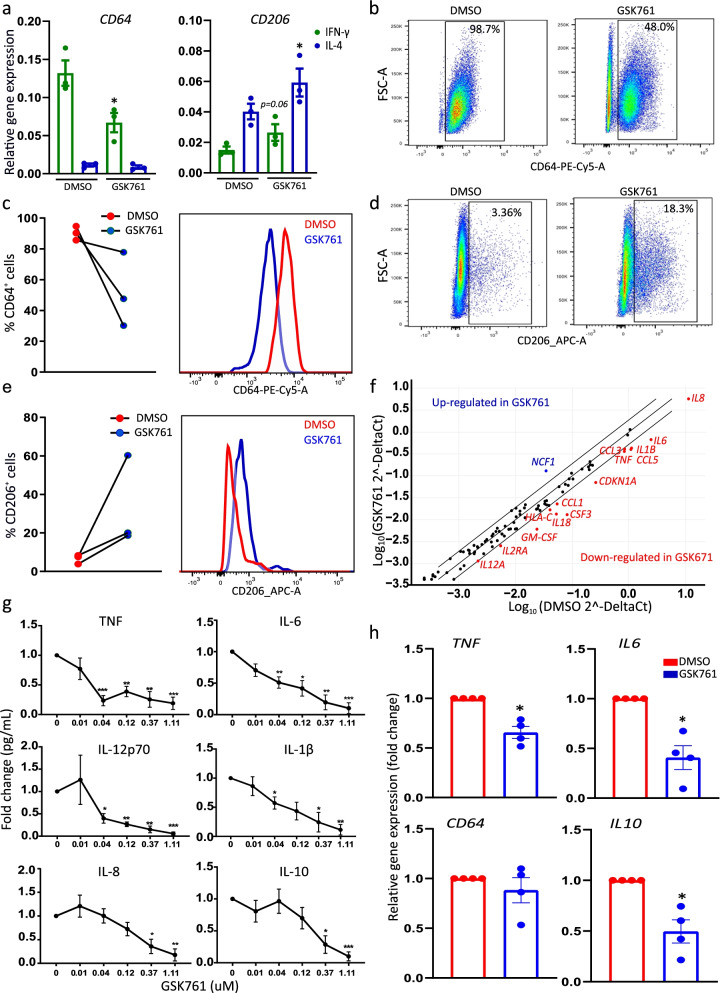
GSK761 affects macrophage polarization and cytokine production in peripheral blood and CD mucosal macrophages. Human primary CD14^+^ monocytes were differentiated with 20 ng/mL M-CSF for 3 days. The cells where then washed with PBS and treated with 0.1% DMSO or 0.04 μM GSK761 for 1 h prior to 3 days polarization to “M1” and “M2” macrophages phenotypes with 100 ng/mL INF-γ or 40 ng/mL IL-4, respectively (GSK761 and DMSO were not washed and kept in the culture during the 3 days of polarization). **a** Gene expression of the inflammatory marker CD64 (“M1” marker) and anti-inflammatory marker CD206 (“M2” marker) was measured by qPCR in IFN-γ (“M1”) and IL-4 (“M2”) polarized macrophages and **b–e** FACS analysis was performed in IFN-γ (“M1”) polarized macrophages. **b** Scatter plots illustrating the frequency of CD64^+^ cells. **c** A graph bar summarizing the frequency of CD64^+^ macrophages in 3 donors (left) and CD64 protein expression intensity (right). **d** Scatter plots illustrating the frequency of CD206^+^ cells. **e** A graph bar summarizing the frequency of CD206^+^ macrophages in 3 donors (left) and CD206 protein expression intensity, *n*=3. **f** “M1” polarized macrophages were pretreated for 1 h with 1% DMSO (*n*=4) or with 0.04 μM GSK761 (*n*=5). The cells were then stimulated for 4 h with 100 ng/mL LPS and Customized RT^2^ Profiler PCR Arrays was performed, the scatter plot illustrates the differentially expressed genes (2-fold change). **g** “M1” polarized macrophages were pretreated for 1 h with 1% DMSO or with an increasing concentration of GSK761 (0.01, 0.04, 0.12, 0.37, and 1.11 μM). The cells were then stimulated for 24 h with 100 ng/mL LPS. Protein levels of TNF, IL-6, IL-1β, IL-10, IL-8, and IL-12p70 were measured in the supernatant, *n*=4–7. The *Y* axis indicates the fold change in cytokine protein expression relative to DMSO control. **h** CD14^+^ cells were isolated from inflamed CD mucosa. The cells were then incubated ex vivo for 4 h with either 0.1% DMSO or 0.04 μM GSK761. Relative gene expression of *TNF*, *IL6*, *IL10*, and *CD64* were measured using qPCR, *n*=4. The *Y* axis indicates the fold change in mRNA level relative to DMSO control. Statistical significance is indicated as follows: *P < 0.05, **P < 0.01, ***P < 0.001, ****P < 0.0001

SP140 expression was selectively increased in “M1” compared to “M0” macrophages, prompting us to test whether SP140 is required for the polarization to an inflammatory macrophage phenotype. We first tested whether GSK761 demonstrated any toxicity in macrophages to determine the appropriate dose range. At ≤0.12 μM, GSK761 showed no cytotoxicity (Additional file 1: Figs. S5a and b). To investigate the effect of GSK761 on macrophage polarization to inflammatory phenotype, “M0” macrophages were treated with either DMSO or GSK761 for 3 days in presence of IFN-γ or IL-4 (during the polarization to “M1” and “M2” macrophages, respectively). GSK761 enhanced mRNA expression of the anti-inflammatory marker *CD206* in both “M1” and “M2” macrophages and decreased the pro-inflammatory marker *CD64* in “M1” macrophages (Fig. 5a). FACS analysis showed that GSK761-treatment prior to IFN-γ (“M1”) polarization reduced CD64^+^ cells and CD64 protein expression (Fig. 5b, c) and increased CD206^+^ cells and CD206 protein expression (Fig. 5d, e). Adding GSK761 to the cells during IFN-γ (“M1”) polarization lowered *TNF* (“M1” polarization marker) gene expression in these cells compared to the control (Additional file 1: Fig. S5d). Altogether, these data suggest that SP140 inhibition during “M1” polarization biased differentiation towards a regulatory “M2” phenotype.

We next assessed the effect of SP140 inhibition on the response of “M1” polarized macrophages to inflammatory stimuli. LPS-stimulated “M1” macrophages pretreated with GSK761 showed a strong reduction in secretion of IL-6, TNF, IL-1β, and IL-12 (Fig. 5g) at concentrations where cytotoxicity was not observed (0.04 and 0.12 μM). When employing RNA transcriptional profiling using a customized qPCR array, SP140 inhibition was found to reduce the expression of many other pro-inflammatory cytokines and chemokines including *GM-CSF*, *CCL3*, *CCL5*, and *CCL1* (Fig. 5f).

The marked anti-inflammatory effects of GSK761 on macrophages in vitro suggest that targeting SP140 may be an effective approach for IBD. Unfortunately, due to poor in vivo pharmokinetics (data not shown), GSK761 was not suitable to evaluate the effects of SP140 inhibition in in vivo animal models of colitis. To evaluate the impact of SP140 inhibition in human CD, CD14^+^ mucosal macrophages were isolated from CD anti-TNF refractory patients’ colonic mucosa and then cultured in vitro with either DMSO or GSK761 for 4 h. Spontaneous gene expression of *TNF*, *IL6*, and *IL10* was significantly decreased in GSK761-treated tissue macrophages, demonstrating that GSK761 inhibited their immune reactivity (Fig. 5h). No changes in *CD64* expression were observed. Previous studies have demonstrated that successful anti-TNF therapy is associated with an increase in CD206^+^ regulatory macrophages [34] and a decrease in pro-inflammatory cytokines [10] and SP140 expression [10] in CD mucosa. Thus, our data suggests that SP140 inhibition could be also beneficial in supporting the anti-TNF therapy. Thus, combining SP140 inhibition with anti-TNF may potentiate the anti-inflammatory effects in macrophages and possibly in IBD in general. Consistent with this idea, M1 macrophages treated in vitro with both GSK761 and Infliximab (anti-TNF) demonstrated an additive reduction in TNF release in response to LPS compared to those treated with GSK761 or infliximab alone (Additional file 1: Fig. S5e).

### SP140 preferentially interacts with transcription start sites (TSS) and enhancer regions

SP140 possesses multiple chromatin binding domains, namely Brd, PHD, and SAND (*S*P100, *A*IRE-1, *N*ucP41/75, *D*EAF-1) domains (Additional file 1: Fig. S1a), which may allow it to function as an epigenetic reader [10, 35]. To assess which histone peptides might be bound by SP140, we utilized an Active Motive histone peptide array, which showed strong binding of SP140 PHD-Brd to unmodified H3_1-19_ and H3_26-45_ peptides, while little binding to unmodified H2A, H2B, and H4 peptides was detected (Additional file 1: Fig. S6a). SP140 PHD-Brd was able to bind several modified H3 peptides bearing some methylation and acetylation marks including K27Ac, K14Ac, K4me3, and K9me3, and also to acetylated H4 peptides (Additional file 1: Fig. S6a).

To measure histone peptide binding in a more quantitative way, and to evaluate peptides for which binding is specifically dependent on the SP140 binding site of GSK761, we tested the ability of a range of peptides to compete for compound binding to SP140 PHD-Brd. We found that recombinant SP140 PHD-Brd protein bound to histone H3 peptides with sub-μM affinity (Additional file 1: Fig. S6b). The strongest binding was observed for an unmodified peptide corresponding to the N-terminal 21 aa, while methylation at lysine residue 4 (K4) led to substantially (~30-fold) reduced affinity (Additional file 1: Fig. S6c). K9 acetylation also resulted in lower affinity binding (4-5-fold), while SP140 binding was unaffected by either acetylation or methylation of K14 (Additional file 1: Fig. S6d). Similar binding affinity was observed for unmodified H3_1-21_ vs H3_1-18_, while reduced (4–5-fold) but still significant SP140 binding was measured for H3_1-9_ (Additional file 1: Fig. S6d). Taken together, these data suggest that the SP140 PHD-Brd module can function as a reader for unmodified histone H3, with binding focused around the N-terminal 9 aa, and that GSK761 competes for this binding.

**Fig. 6:**
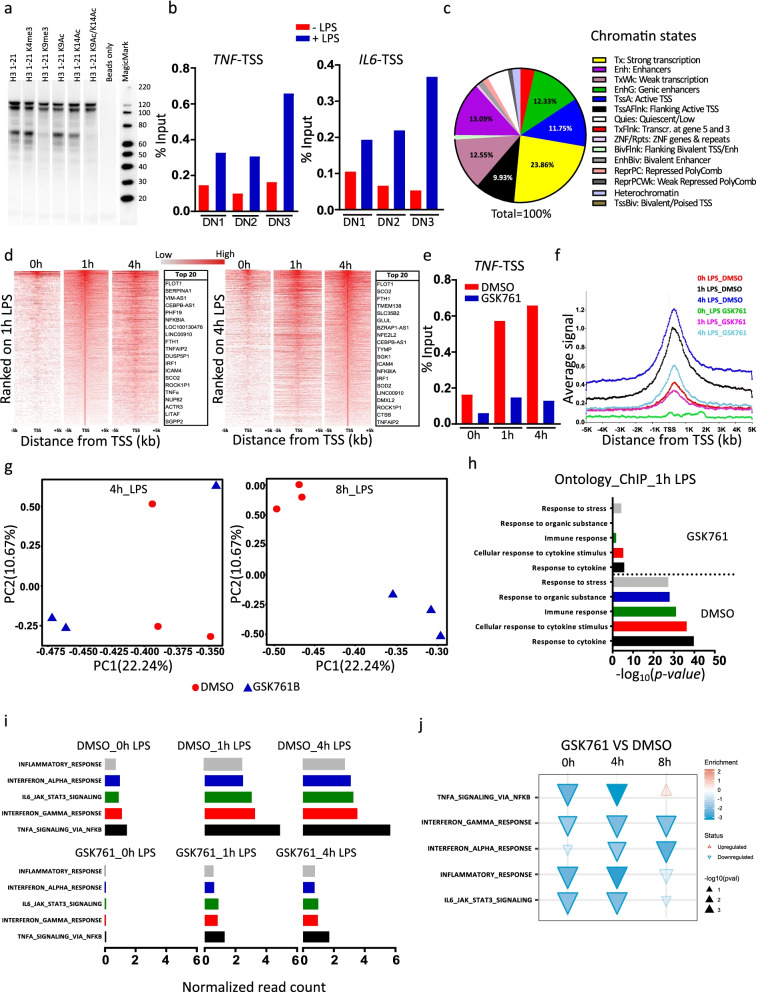
LPS stimulation leads to SP140 protein recruitment to chromatin; GSK761 reduces this recruitment and dampens inflammatory pathways in inflammatory macrophages. **a** Nuclear extracts from αCD3/αCD28 stimulated HuT78 cells were incubated with unmodified or modified (acetylated and methylated) histone H3 peptides as indicated. SP140 was then pulled down to visualize its interaction with H3 peptides. **b** ChIP-qPCR of SP140 occupancy at TSS of *TNF* and *IL6* in “M1” macrophages stimulated with 100 ng/mL LPS for 4 h or without LPS stimulation, *n*=3 donors (DN). **c** Epigenome roadmap scan illustrating proportions of SP140 genome-wide occupancy after SP140 ChIP-seq. **d** Heatmap (1000 top SP140-bound genes) of SP140 ChIP-seq reads ranked on 1 h LPS-stimulated (left) or 4 h LPS-stimulated (right) “M1” macrophages rank-ordered from high to low occupancy centered on TSS. Top 20 genes with high SP140 occupancy are listed. **e** ChIP-qPCR of SP140 occupancy at TSS of *TNF* in “M1” macrophages pretreated with 0.1% DMSO or 0.04 μM GSK761 for 1 h and then stimulated with 100 ng/mL LPS for 1 or 4 h or kept unstimulated (0 h LPS). **f** Metagene created from normalized genome-wide average reads for SP140 centered on TSS. **g** PCA of RNA-seq comparing 0.1% DMSO to 0.04 μM GSK761 treated “M1” macrophages after 4 h of LPS stimulation (left) or after 8 h of LPS stimulation (right). **h** SP140 ChIP-seq gene ontology analysis of the most enriched molecular function and biological process after 1 h of LPS stimulation, comparing 0.1% DMSO with 0.04 μM GSK761 treated “M1” macrophages. **i** Hallmarks pathway enrichment analysis at 0, 1, and 4 h of 100 ng/mL LPS stimulation for ChIP-seq and at **j** 0, 4, and 8 h of 100 ng/mL LPS stimulation for RNA-seq. **j** The direction and color of the arrow indicate the direction and size of the enrichment score, the size of the arrow is proportional to the −log_10_ (*p*-value), and non-transparent arrows represent significantly affected pathways

To evaluate whether native SP140 is also capable of binding to histones, we used immobilized histone H3 peptides with varying modifications to precipitate proteins from nuclear extracts of anti-CD3/CD28-stimulated HuT78 T cells and probed via Western blotting for the presence of SP140. SP140 was efficiently pulled down by unmodified H3_1-21_ (Fig. 6a). Notably, H3_1-21_ peptides bearing certain methylation and acetylation marks (K4me3, K9me3, K9ac, and K14ac) were also capable of capturing native SP140 (Fig. 6a), despite the lower measured affinity of some of these for recombinant SP140 PHD-Brd (see above). To evaluate whether SP140 interacts with histone H3 in a cellular setting, we utilized a NanoBRET system (Additional file 1: Fig. S6e). NanoBRET is a proximity-based assay that can detect protein interactions by measuring energy transfer from a bioluminescent protein donor NanoLuc® (NL) to a fluorescent protein acceptor HaloTag® (HT). This energy transfer was observed in the nuclei of HEK293 cells transfected with SP140-NL and Histone3.3-HT DNA, indicating close proximity of the two proteins (Additional file 1: Fig. S6e).

To assess whether SP140 associates with chromatin in macrophages and how this might be regulated in inflammation, we initially conducted ChIP-qPCR experiments in unstimulated or LPS-stimulated “M1” macrophages. First, the specificity of the antibody used for ChIP experiments to precipitate SP140 protein was verified (Additional file 1: Fig. S7a). Binding of SP140 to the TSS of *TNF* and *IL6* genes was observed in unstimulated cells, and this was increased following LPS stimulation (Fig. 6b). We then evaluated chromatin occupancy of SP140 on a genome-wide level using ChIP-seq and tested the effects of GSK761 on both SP140 binding and gene expression (RNA-seq) in the context of LPS stimulation. In DMSO-treated, LPS-stimulated macrophages, epigenome roadmap scan analysis revealed that the majority of SP140 occupancy was at strong transcription associated regions, enhancers, and TSS regions (Fig. 6c). However, this occupancy was decreased when the cells were pretreated with GSK761 at 1 h (Additional file 1: Fig. S7c) and 4 h (data not shown) post LPS stimulation. Heatmap rank ordering of SP140 occupancy in unstimulated and LPS-stimulated “M1” macrophages showed a strong SP140 enrichment at the TSS (Fig. 6d). The top 20 enriched genes belonged mostly to those involved in the innate immune response, including *TNF*, *ICAM4*, *IRF1*, *LITAF*, *TNFAIP2*, and *NFKBIA* (Fig. 6d). This was confirmed when Metagene analysis showed that SP140 preferentially binds near the TSS of immune innate genes, while this binding was minimal at the TSS of non-immune genes (Additional file 1: Fig. S7b). However, the most SP140-enriched gene *FLOT1* (Fig. 6d) has been reported to be strongly involved in tumorigenesis [36] and anti-fungal immunity [37]. We found SP140 also to occupy active enhancers, as marked by H3K27Ac in human macrophages by overlapping the publicly available H3K27Ac ChIP-seq data (GSE54972) with our SP140 ChIP-seq dataset at 1 h of LPS stimulation (Additional file 1: Fig. S7d). ChIP-qPCR showed a strong reduction of SP140 occupancy at the *TNF*-TSS in GSK761-treated “M1” macrophages, which was reduced to that of unstimulated cells (Fig. 6e), correlating with the previously observed reduced expression of TNF in GSK761-treated macrophages.

Following LPS stimulation, we observed a marked increase in binding at the TSS of the top 1000 genes occupied by SP140, reaching its maximum at 4 h (Fig. 6f). GSK761 treatment prior to LPS stimulation strongly reduced SP140 occupancy at the TSS (Fig. 6f) associated with an altered LPS-induced gene expression (Fig. 6g). PCA of RNA-seq data revealed a large separation in global gene expression between DMSO- and GSK761-treated macrophages at both time points post of LPS stimulation (4h and 8h) (Fig. 6g). Note that *SP140* itself did not appear to be bound by SP140 protein, nor was its expression altered by GSK761 treatment (Additional files 5, 6 and 7: Table S5-7 and Additional files 12 and 13: Table S12-13).

Gene Ontology analysis of SP140-bound genes showed enrichment of genes that participate in the processes of cytokine response and immune response that were inhibited through GSK761 pre-exposure (Fig. 6h). To elucidate the regulatory pathways enriched by SP140 and affected by GSK761, we carried out hallmark pathway analysis for SP140 differentially bound genes (DBGs) (Fig. 6i) and DEGs (Fig. 6j) upon DMSO or GSK761 treatment at each time point of LPS stimulation. In DMSO-treated “M1” macrophages, we found that SP140 binding is predominantly enriched at many pathways typically defined as inflammatory such as TNF signaling via NFKB, and inflammatory response (Fig. 6i). However, this enrichment was no longer seen in GSK761-treated macrophages (Fig. 6i). Similar pathways were observed for DEGs and were downregulated in GSK761-treated macrophages, especially at 4 h (Fig. 6j). In addition, we observed an upregulation of MYC targets and oxidative phosphorylation gene sets in GSK761-treated macrophages, whereas these normally are downregulated because of the induction of aerobic glycolysis upon LPS stimulation of macrophages (Additional file 1: Fig. S8a) [38]. These data suggest SP140 as a critical regulator of genes involved in the inflammatory response and that GSK761 can inhibit this response through displacing SP140 binding to TSS and enhancer regions.

### SP140 preferentially controls the expression of specific gene sets involved in the innate immune response

**Fig. 7:**
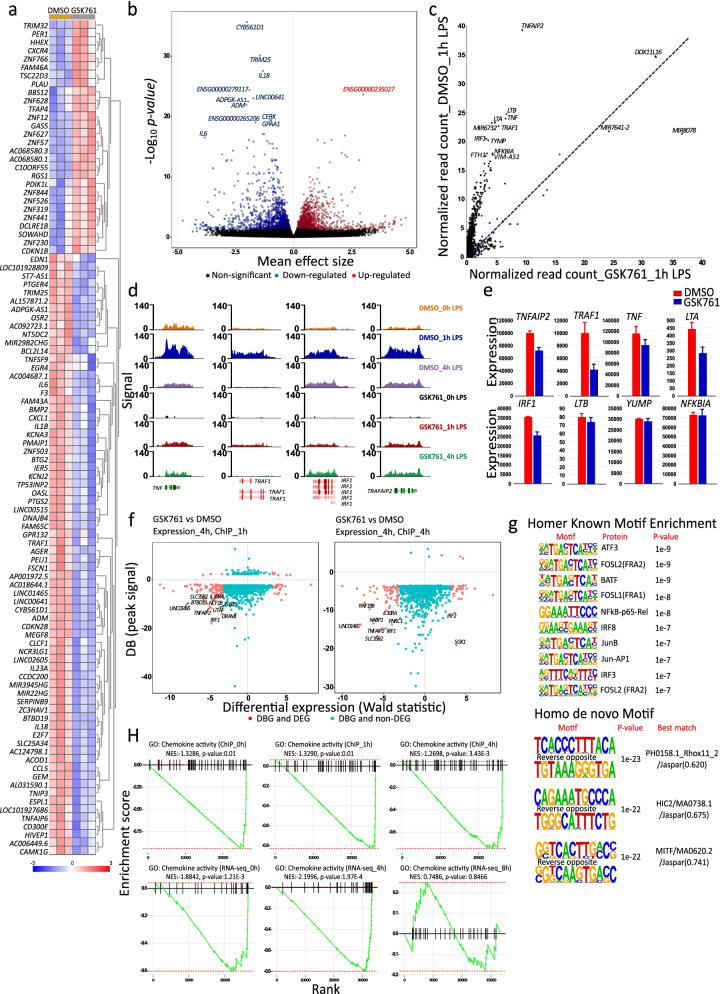
SP140 preferentially controls the expression of specific gene sets involved in the innate immune response. **a** Heatmap of the top 100 DEGs and **b** volcano plots of the all genes comparing 0.1% DMSO- with 0.04 μM GSK761-treated “M1” macrophages after 4 h of 100 ng/mL LPS stimulation. **c** R2 TSS plot comparing a global differentially SP140-bound genes (DBGs) after 1 h of 100 ng/mL LPS stimulation of 0.1% DMSO- and 0.04 μM GSK761-treated “M1” macrophages. **d** SP140 ChIP-seq genome browser view of some of the most affected DBGs; *TNF*, *TRAF1*, *IRF1*, and *TRAFAIP2*. *Y* axis represents a signal score of recovered sequences in 0.1% DMSO and 0.04 μM GSK761 treated macrophages after 0, 1, and 4 h of 100 ng/mL LPS stimulation. **e** RNA sequencing-derived gene expression of some of the most DBGs. **f** Comparative analyses of the top 1000 DBGs (signal) with their gene expression (Wald statistic). Gene expression at 4 h and ChIP at 1 h (left) and gene expression at 4 h and ChIP at 4 h (right), *Y* axis represents the SP140 differential binding signal (BD). **g** Homer Known Motif Enrichment using TF motifs and their respective *p*-value scoring (top) and Homer de novo Motif results with best match TFs (bottom) in 0.1% DMSO-treated “M1” macrophages after 1 h of 100 ng/mL LPS stimulation. **h** An enrichment analysis targeted chemokine activity for SP140 ChIP-seq (top) and RNA-seq (bottom) comparing 0.1% DMSO- with 0.04 μM GSK761-treated “M1” macrophages at 0, 1, and 4 h of 100 ng/mL LPS stimulation

We next investigated the functional significance of inhibiting SP140 binding for gene expression. Heatmap (Top 100) and volcano plots of DEGs after 4 h LPS stimulation demonstrated a strong effect of GSK761 on gene sets that are involved in the innate immune response, such as the downregulation of *TNFSF9*, *IL6*, *F3*, *CXCL1*, *CCL5*, *IL1β*, *TRAF1*, *IL23A*, *IL18*, and *GEM* and the upregulation of *PLAU* and *CXCR4* (Fig. 7a, b). We next explored the global DBGs in 1 h LPS-stimulated “M1” macrophages using R2 TSS plot (Fig. 7c) and R2 TSS-peak calling (Fig. 7d). Interestingly, the top DBGs belonged to TNF signaling via NFKB such as *TNFAIP2*, *TNF*, *LTA*, *TRAF1*, *IRF1*, and *NFKBIA* (Fig. 7c, d). GSK761 clearly reduced SP140 binding to those genes (Fig. 7d). Similarly, gene expression of *TNFAIP2*, *TAF1*, *TNF*, *LTA*, and *IRF1* was reduced (Fig. 7e). By conducting a focused TNF signaling enrichment analysis (Additional file 1: Fig. S8b) and R2 TSS plot analysis (Additional file 1: Fig. S8c), we illustrated a new set of DBGs including TFs (*TNFAIP3*, *CEBPB*, *ATF4*, *MAP3K8*, *MAP2K3*, and *JUNB*), cytokines (*IL1β*, *IL23A*), and adhesion molecules (*ICAM1*). Most of these genes were DEGs (Additional file 1: Fig. S8d).

We next verified the impact of global reduced SP140 binding on gene expression by integrating the DBGs and the DEGs. Interrogating the top 1000 DBGs (at 1 or 4 h LPS) for their gene expression (at 4 or 8 h LPS) implicated genes that are involved in TNF signaling pathway (*NFKBIA*, *SOD2*, *LITAF*, *SGK1*, *PNRC1*, *IRF1*, and *RNF19B*) and cytokine-mediated signaling pathway (For example: *IL10RA*, *NFKBIA*, *IRF1*, *SOD2*, *CAMK2D*, *IRF2*, and *ISG15*) which showed the strongest concordant differential binding and expression (Fig. 7f and S9).

In addition to SP140 binding to chromatin, we speculated that SP140 may also interact with other TFs proteins to regulate gene expression. To this end, we performed homer known motif enrichment analysis (HKMEA) at DMSO_1h LPS (Fig. 7g). We also conducted a homo de novo motif discovery to define new DNA sequence motifs that are bound by SP140 (Fig. 7g). Interestingly, HKMEA suggests that SP140 may bind the same DNA sequence motifs recognized by certain TFs defined as key proteins in TNF signaling via NFKB (ATF3, FOSL2, FOSL1, NFKB-p65-rel, JUNB, and JUN-AP1) and of cytokine-mediated signaling pathway (BATF, NFKB-p65-rel, JUNB, IRF8, and IRF3) (Fig. 7g).

LPS stimulation strongly recruits SP140 to multiple human leukocyte antigen (HLA) genes, including *HLA-A*, *HLA-B*, *HLA-C*, *HLA-F*, *HLA-DPA*, and *HLA-DPB* (Additional file 1: Fig. S8e), binding which was strongly reduced by GSK761. This indicates a role of SP140 in regulating antigen presentation-associated genes. Notably, the Brd-containing protein 2 (*BRD2*) gene was occupied by SP140, and GSK761 reduced this binding and lowered BRD2 gene expression (Additional file 1: Fig. S8e and Additional file 5: Table S5). BRD2 has been reported to play a key role in inflammatory response in murine macrophages and in inducing insulin resistance [39].

Finally, we noticed that SP140 occupancy on TSS of chemokine activity genes was dramatically reduced by GSK761, inducing a reduced global chemokine activity at mRNA level (Fig. 7h). R2 TSS plots and R2 TSS-peak calling indicate a strong SP140 occupancy at several inflammatory macrophage-associated chemokines (*CCL2*, *CCL3*, *CCL4*, *CCL5*, *CCL8*, *CXCL1*, *CXCL3*, *CXCL8 CXCL9*, *CXCL10*, and *CXCL11*), chemokine ligands (*CCL4L1*, *CCL3L1*, *CCL3L3*, *CCL4L2*), chemokine receptors (*CCR7*), TFs (*STAT1*, *JAK2*, *PIK3R5*, *RELA*), and protein kinases (*PRKCD*, *FGR*, and *HCR*) (Additional file 1: Figs. S10a,b and c). This binding was reduced by GSK761, affecting expression of many genes involved in chemokine signaling (Additional file 1: Fig. S10d). However, SP140 binding was selective as it did not bind to the TSS of a range of other chemokines such as *CXCL6*, *CXCL5*, *CCL11*, and *CCL7* (Additional file 1: Fig. S10c).

## Discussion

SP140 has been implicated in CD and other autoimmune diseases through genetic and epigenetic association studies [16, 40]. Here, we describe the first small molecule inhibitor of SP140 protein, which has been used to investigate its function. The novel SP140-binding small molecule GSK761 was shown to compete with the N-terminal tail of histone H3 for interactions with the SP140 PHD-Brd module. In human macrophages, SP140 associated with the regulatory regions of immune-related/inflammatory genes in a stimulus-dependent manner. GSK761 inhibited SP140 binding to the TSS of many inflammatory genes, correlating with compound-induced decreased expression of these genes and reduced macrophage inflammatory function. The predominant SP140 expression in immune cells and in particular inflammatory macrophages, further identifies SP140 as an epigenetic reader protein that contributes to inflammatory gene expression.

Inflammatory macrophages generated in vitro or macrophages from CD inflamed tissue were shown to express high levels of SP140, and *SP140* silencing in macrophages reduced the expression of a range of inflammatory genes. These anti-inflammatory effects were extended using GSK761, which was able to inhibit spontaneous cytokine expression from CD14^+^ macrophages isolated from CD anti-TNF refractory patients’ colonic mucosa. LPS stimulation of macrophages was shown to cause SP140 recruitment to the TSS of a specific set of inflammatory genes, which was prevented by GSK761, dampening the induced expression of those genes. The strongest SP140 occupancy was at the TSS of a range of genes involved in TNF signaling. TNF signaling plays an integral role in CD as evidenced by the efficacy of chimeric anti-TNF mAbs therapy [41]. Notably, anti-TNF therapy response rests on TNF neutralization, but specifically in CD also on its potency to bind membrane-bound TNF on CD tissue infiltrated macrophages [42], which causes differentiation of macrophages to a more regulatory CD206^+^ “M2”-like phenotype [34, 43]. This is for example evidenced by the clinical observation using etanercept (does not bind membrane-bound TNF), which is not effective in CD, while infliximab is [43]. A reduced expression of SP140 in CD biopsies was shown to be correlated with a better anti-TNF response [10]. In this study, we found that GSK761 increased CD206 expressing macrophages in in vitro*.* Furthermore, GSK761 reduced pro-inflammatory cytokine expression in CD mucosal macrophages of anti-TNF-resistant patients. Interestingly, the combination of GSK761 and infliximab was more effective at reducing TNF protein secretion in inflammatory macrophages in vitro than either treatment alone. Thus, we anticipate that SP140 inhibition would be useful in enhancing anti-TNF remission induction in CD patients. In this respect, SP140 was also shown by ChIP-seq analysis to bind and regulate expression of some key CD-associated genes such as *IL23A* [44], *TYK2* [27, 28], *JAK2* [27, 28], *NOD2* [45], and *CARD9* [46].

In IBD, epigenetic mechanisms control macrophage inflammatory activation and polarization [47]. These macrophages are central components of the inflamed mucosa and contribute to disease pathology by producing inflammatory cytokines, which promote the differentiation and activation of Th1 and Th17 cells [48]. In this study, SP140 is shown to be critical for both the polarization of inflammatory macrophages and in the response of polarized macrophages to inflammatory stimuli. In particular, the TFs STAT1 and STAT2 are crucial for inflammatory macrophage polarization [5, 49, 50]. We found high levels of SP140 binding associated with the *STAT1* and *STAT2* genes; GSK761 disrupted SP140 binding to these genes and reduced their expression, providing a probable mechanistic basis for the inhibitory effects of the compound on “M1” polarization. Notably, GSK761 inhibited macrophage-induced cytokines and costimulatory molecules required for inflammatory T cell activation including *IL23A* [44], *IL12* [51], *IL18* [52], *IL1β* [52], *CD40* [53], and *CD80* [54]*.* Therefore, in addition to reducing the direct inflammatory function of macrophages, inhibition of SP140 may inhibit downstream activation/polarization of responding T cells.

While the mechanism by which SP140 is targeted to gene regulatory regions remains to be determined, motif analysis indicated a strong overlap between the DNA sequences enriched for SP140 binding and the binding motifs of some key TFs involved in regulating the inflammatory response as well as the differentiation and polarization to inflammatory macrophages, such as NFkB-p65-rel [55], IRF3 [56], and IRF8 [57]. Thus, it is possible that SP140 is recruited to chromatin via binding to stimulation-induced TFs—either before or after the TFs bind to DNA. Conversely, SP140 may associate with other proteins recruited to sites bound by these TFs. By conducting ChIP-seq at multiple time points (1 and 4 h), we observed differential binding kinetics of SP140 to different sets of genes; for example, SP140 is bound to the *CCL5* TSS by 1 h after LPS stimulation but the TSS of *CCL2* only after 4 h. The factors controlling the differential binding kinetics are currently unknown but could include differences in the initial chromatin environments of these genes in addition to distinct kinetics of TF activation and binding.

In a previous study, SP140 was found to be enriched at the *HOXA* genes in macrophages, and SP140 binding at these locations was proposed to play a role in repression of these lineage-inappropriate genes [10]. However, SP140 binding to this region was minimal in our study in both unstimulated and LPS-stimulated macrophages (Additional files 8, 9 and 10: Tables S8-10). In addition, GSK761 did not affect *HOXA* genes expression in M1 macrophages (Additional files 5, 6 and 7: Tables S5-7). Based on our findings and contrary to the observation of Mehta et al., *HOXA* genes do not appear to be bound by SP140, nor does their expression seem to be controlled by SP140. The reason for this discrepancy is unclear, but the use of different SP140 antibodies in the two studies is one possibility. The antibody used in our ChIP experiments was found to be the most SP140-specific among 5 antibodies that we tested. Furthermore, our ChIP-seq data has demonstrated a strong association between SP140 and several enhancer/strong transcription regions (Fig. 6c). GSK761 was able to reduce SP140 binding to those regions and as a consequence the global gene expression as represented in PCA in Fig. 6g. The displacement of SP140 binding by GSK761 provides strong support for the specificity of the SP140 binding identified in our ChIP-seq study. Altogether, we should conclude from our data that SP140 functions as a gene expression activator rather than a repressor.

Analysis of the gene sets identified in our SP140 ChIP-seq study suggested a role for SP140 in immune defense against microbes (in accordance with [58] and [59]) and some other diseases, such as graft-versus-host disease, cancers, and rheumatoid arthritis. The intestinal microbiota is thought to play a central role in the pathogenesis of CD, and subsequent investigations could consider the role of SP140 in this respect as a previous study linked *SP140* SNPs to microbial dysbiosis in human intestinal microbiota [60]. In this study, ChIP-seq analysis was mainly focused on investigating SP140 binding to coding genes. However, by including non-coding RNA molecules, we observed very strong SP140 binding to a limited set of microRNAs (miRNAs) and long non-coding RNAs (lncRNAs) mainly involved in tumorigenesis, such as the miRNAs: MIR3687 [61], MIR3648 [62], and MIR663A [63], and the IncRNAs: MALAT1 [64] and NEAT1 [64]. Accordingly, previous studies have demonstrated that certain BCPs (such as BRD4) are capable of binding and modulating the transcription of some miRNAs and IncRNAs involved in tumorogenesis [65–67]. Contrary to the coding genes and the IncRNAs, GSK761 was unable to affect SP140 binding to miRNAs. This suggests that SP140 binding in these locations is independent of the PHD-Brd region with which GSK761 interacts; binding of SP140 to DNA via the SAND domain is one possibility. While not the focus of this study, other potential links between SP140 and cancer were observed, including high expression of SP140 in chronic lymphocytic leukemia patients’ cells (although reduced expression in acute myelogenous leukemia), and high binding of SP140 to the *FLOT1* gene, which has been implicated in tumorigenesis [36]

## Conclusions

We conclude that SP140 is an epigenetic reader protein that regulates gene expression in macrophages in response to inflammatory stimuli, including those present in the CD intestinal mucosa of patients failing therapy. Targeting SP140 with a selective inhibitor was shown to displace SP140 from chromatin and decrease inflammatory gene expression in macrophages. This study suggests that SP140 inhibition could represent a promising approach for CD, which might include in addition to remission induction, the potential for combination with anti-TNF therapy.

## Methods

### In-house GSK microarray profiler

Human gene expression data, along with accompanying sample descriptions, was purchased from GeneLogic (GeneLogic Division, Ocimum Biosolutions, Inc.) in 2006, and later organized by sample type. Gene expression data for each sample had been determined using mRNA amplification protocols as recommended by Affymetrix (Affymetrix, Inc.) and subsequently hybridized to the Affymetrix U133_plus2 chip. Purchased data was subject to reported quality control measures including ratios for *ACTB* and *GAPDH*, as well as maximal scale factors as reported by Affymetrix MAS 5.0. Expression data was normalized using MAS5.0 with a target intensity of 150.

### Immunohistochemistry

An anti-SP140 antibody produced in rabbit (HPA006162; Sigma Prestige Antibody) was used to visualize SP140 protein in a Cambridge Bioscience 69571061 Normal Human tissue microarray, a Cambridge Bioscience 4013301 Human Autoimmune Array, a Cambridge Bioscience 4013101 Human Colitis Array, and on in-house rheumatoid arthritis synovial samples. Anti-SP140 antibody was detected with a Leica polymer secondary antibody. Sections were de-waxed using proprietary ER1, low pH 6 and ER2, high pH 8 buffers for 20 min at 98 °C. Sites of antibody binding were visualized with peroxidase and DAB. Staining was performed on a Leica Bondman immunostaining instrument, using protocol IHC F.

### Tissue immunofluorescence of SP140, CD68, and HLA-DR

Paraffin-embedded tissue sections from inflamed and non-inflamed colon were obtained from patients with CD during surgical procedures, were first deparaffinized and washed with TBS. For antigen retrieval, slides were treated at 96 °C for 10 min in 0.01 M sodium citrate buffer pH 6.0. The tissue sections were blocked and permeabilized with PBT (PBS, 0.1% Triton X-100 (Biorad), 1% w/v BSA (Sigma-Aldrich)) and incubated with primary antibodies for 2 h (h) at room temperature (RT), to detect pan-macrophage marker CD68 (dilution 1:200) (M0876, Dako), SP140 (dilution 1:200) (ab171141; Abcam) and mouse anti-human HLA-DR (dilution 1:100) (Becton Dickinson). After washing, slides were stained with the following secondary antibodies: polyclonal goat anti-Mouse Alexa Fluor488 to detect CD68 or HLA-DR protein (Green color) (A-11029, Invitrogen) or polyclonal goat anti-Rabbit Alexa Fluor546 to detect SP140 protein (Orange color) (A-11035, Invitrogen). Slides were mounted in SlowfadeGold reagent containing DAPI (4′,6-diamidino-2-phenylindole, Thermo Fisher) and imaged using a Leica DM6000B microscope equipped with LAS-X software (Leica Microsystems).

### SP140 expression in ileal macrophages

Publicly available single-cell RNA sequencing data from 11 involved (inflamed) and 11 paired uninvolved (uninflamed) ileal biopsies was downloaded from the sequence read archive accession number SRP216273 [24]. Raw reads were aligned against GRCh38 using Cellranger (v3.1.0). The resulting unique molecular identifier (UMI) count matrices were then imported into the R statistical environment (v3.6.3) whereupon the samples were analyzed in an integrative fashion using Seurat (v3.1.5) [68, 69]. Clustering analyses were performed using the top 2000 most variable genes and the top 15 principal components yielding 22 clusters whereupon they were visualized through UMAP (arXiv:1802.03426). Clusters 8, 11, 13, 14, and 18 were annotated as epithelial cells (*PTPRC*−*VIL1*+*CDH1*+). Cluster 20 was annotated as the glial cells (*PTPRC*−*ERBB3*+*PLP1*+*S100B*+). Cluster 15 was annotated as the fibroblasts (*PTPRC*−*LUM*+*COL1A1*+*COL1A2*+). Cluster 16 was annotated as the endothelial cells (*PTPRC−FLT1+ICAM1+*). Clusters 0, 2, 3, 4, 5, 6, 7, 9, 10, 17, 19, and 21 were annotated as immune cells (*PTPRC*+). Cluster 0 was identified as the B cells (*PTPRC+MS4A1+CD19+CD27+JCHAIN−*), Clusters 3, 9, and 12 were annotated as the plasma cells (*PTPRC−MS4A1−CD19−CD27+JCHAIN+*). Cluster 1, 5, and 10 were annotated as the CD4 T cells (*PTPRC+CD3D+CD4+CD8−*). Clusters 2 and 4 were annotated as the CD8 T cells (*PTPRC+CD3D+CD4−CD8+*). Cluster 6 was annotated as the mononuclear phagocytes (MNP) (*PTPRC+CD68+FCER1A+CD163+MRC1+CD14*+*FCGR3A+*). Cluster 7 was annotated as the natural killer (NK) cells (PTPRC+CD8A+NKG7+NCAM1+). Cluster 17 was annotated as the type 2 innate lymphoid cells (*PTPRC+AREG+IL7R+KLRB1+*). Cluster 19 was annotated as mast cells (*PTPRC+KIT+TPSB2+TPSAB1+ITGAM+*). Cluster 21 was annotated as the plasmacytoid dendritic cells (pDC) (*PTPRC+CD1C+IRF4+IRF8+HLA−DPA1+IL3RA+*). To further disentangle the MNPs, we redid the clustering and classification analysis on cluster 6 alone, which yielded 10 subclusters. Clusters 6.2, 6.4, and 6.8 were annotated as monocytes (*CD14+FCGR3A+*). Cluster 6.0 was annotated as the dendritic cells (DC) (*CD1C+CLEC4A+HLA−DQA1+*). Clusters 6.5 and 6.6 were annotated as macrophages (*CD168+FCER1A+MRC1+*), which were notably inflammatory (*TNF+IL6+*). Cluster 6.1 and 6.3 were dead or dying DCs and macrophages, respectively as they presented a high percentage of mitochondrial reads and a low number of unique genes per cell. Cluster 6.9 was a dead or dying cell population as well but could not be annotated to any of the MNPs. Similarly, cluster 6.7 did not fit any of the known MNPs. The monocyte development trajectories were inferred using slingshot (v1.6.1) [70]. Differential abundance analyses were performed using the Wald test as implemented in DESeq2 (v1.24.0). Comparative percentage non-zero cells were performed using *t*-tests.

### Isolation, differentiation, and polarization of primary human monocytes and THP-1 cells

Peripheral blood mononuclear cells (PBMCs) were obtained from whole blood of healthy donors (from Sanquin Institute Amsterdam or from GSK Stevenage Blood Donation Unit) by Ficoll density gradient (Invitrogen). CD14^+^ monocytes were positively selected from PBMCs using CD14 Microbeads according to the manufacturer’s instructions (Miltenyi Biotec). CD14^+^ cells were differentiated with 20 ng/mL of macrophage colony-stimulating factor (M-CSF) (R&D systems) for 3 days followed by 3 days of polarization into naïve macrophages (“M0”) (media only) [71], classically activated (inflammatory) “M1” macrophages (100 ng/mL IFN-γ; R&D systems) [71], or alternative activated (regulatory) “M2” macrophages (40 ng/mL IL4;R&D systems) [71]. Human monocytic cell line (THP-1) cells were differentiated with 100 nM phorbol myristate acetate (Sigma-Aldrich) for 3 days and then the same polarization protocol was performed. Cells were incubated in Isocove’s modified Dulbecco’s medium (Lonza) supplemented with 10% fetal bovine serum (FBS) (Lonza), 2 mM l-glutamine (Lonza), 100 U/mL penicillin (Lonza), and 100 U/mL streptomycin (Lonza), at 37 °C, 5 % CO_2_.

### siRNA-mediated SP140 knockdown

Human “M1” macrophages were generated in vitro from human primary CD14^+^ monocytes as described above. “M1” macrophages were transfected with siGENOME human smartpool *SP140* siRNA or non-targeting scrambled siRNA for 48 h with DharmaFECT™ transfection reagents according to manufacturer’s protocol (Dharmacon). The cells were left unstimulated or stimulated with 100 ng/mL LPS (*E. coli* 0111:B4; Sigma) for 4 h (for qPCR) or 24 h (for Elisa). The supernatant was harvested for cytokine measurement and the cells were lysed (ISOLATE II RNA Lysis Buffer RLY- Bioline) for RNA extraction.

### RNA isolation and reverse transcriptase, polymerase chain reaction (PCR), and real-time quantitative PCR

Total RNA was extracted from macrophages using RNeasy Mini Kit (Qiagen) following the manufacturer’s instructions. The concentration of RNA was determined using spectrophotometry (Nano-Drop ND-1000). Complementary DNA (cDNA) was synthesized with qScript cDNA SuperMix (Quanta Biosciences). PCR amplification of *SP140*, *SP100*, *SP110*, *SP140L*, *BRDT*, *BRD2*, *BRD3*, *BRD4*, *BRD9*, *EP300*, *BAZ2A*, *BAZ2B*, *PCAF*, *CREBBP*, *TNF*, *IL6*, *IL10*, *IL8*, *CCL5*, *CCL22*, *CD206*, and *CD64* was performed by Fast Start DNA Master^plus^ SYBR Green I kit on the Light Cycler 480 (Roche, Applied Science). Relative RNA expression levels were normalized to the geometric mean of two reference genes *RPL37A* and *ACTB*. Primer sequences are listed in Additional file 4: Table S3.

### Immunofluorescence cell staining

Primary human monocyte-derived macrophages were polarized to “M1” phenotype on coverslips. The cells were fixed with 4% paraformaldehyde and then permeabilized in 0.2% Triton (Biorad)/PBS. Blocking buffer 2% BSA (Sigma-Aldrich) was added for 30 min. Rabbit polyclonal anti-SP140 antibody (dilution 1:200) (ab171141; Abcam) or mouse polyclonal anti-SP100 antibody (dilution 1:200) (ab167605; Abcam) were added for 2 h) followed by 2 h of secondary antibody, Polyclonal goat anti-Rabbit, Alexa Fluor546 (A-11035, Invitrogen) (dilution of 1:1000), or goat anti-mouse, Alexa Fluor546 (A-21123, Life Technologies) (dilution of 1:500), respectively. DAPI (Thermo Fisher) was used for nuclear detection.

### Microarray

RNA from human “M1” macrophages transfected with *SP140* siRNA or scrambled siRNA was extracted using a RNeasy mini kit (Qiagen). One hundred fifty nanograms total RNA was labelled using the cRNA labelling kit for Illumina BeadArrays (Ambion) and hybridized with Ref8v3 BeadArrays (Illumina). Arrays were scanned on a BeadArray 500GX scanner, and data were normalized using quantile normalization with background subtraction (GenomeStudio software; Illumina). Genes with negative values were removed from the analysis. Differentially expressed genes (DEGs) had a *P*-value <0.05 (analysis of variance). The data were analyzed in R (v3.6.3) and R2 Genomics Analysis and Visualization Platform-UMC (r2.amc.nl). Gene ontology overrepresentation analyses were performed in ShinyGO v0.60 [72].

### Discovery and synthesis of the compounds

#### The development of a selective inhibitor of SP140 (GSK761)

To identify selective SP140 binding compounds, we utilized encoded library technology to screen the GSK proprietary collection of DNA-encoded small molecule libraries (DELs) of more than 1 billion unique molecules [31, 32]. Affinity selection utilizing a recombinant protein construct spanning the PHD and Brd domains of SP140 was carried out. The SP140 (687-867) protein was first immobilized on an Anti-Flag resin tip (Phynexus), a pool of DEL molecules was passed over the bound protein for 1 h and the non-binders were washed away. The protein was denatured at 80 °C, and the bound library molecules were recovered. Two additional rounds of selection were performed, using fresh protein at each round. In addition, a parallel selection with Anti-Flag resins only was carried as a no-target control to eliminate matrix binders. After three rounds of selection, the DNA tags of the eluted population were PCR amplified and sequenced, and the sequences were translated to identify structures of putative SP140 binders. One disynton series (combination of two building blocks) of putative SP140 binders was identified from a three-cycle benzimidazole library (Fig. 4a). The library was synthesized by first installing the DNA head piece to two BB1 nitrobenzoic acids (i.e., 3-fluoro-4-nitrobenzoic acid and 4-fluoro-3-nitrobenzoic acid) by SNAr reactions. After reduction of the nitro group to an amine group, 1456 BB2 aldehydes were incorporated to the DNA-linked anilines through reductive alkylation. Finally, 3157 BB3 amines were used to cap the free acids via the amide bond formation. This library contains 9.19 million different compounds, each of which is encoded by a unique DNA tag. The analysis of the affinity selection output was performed in a Spotfire cube view with each axis represents a cycle of diversity in the library. In Fig. 4b, individual points, corresponding to discrete small molecule warheads enriched from this benzimidazole library, are shown and sized according to the number of unique instances recorded by DNA sequencing. Families of related compounds are of particular interest because they contain one or more building blocks in common and are easily recognized in these views as lines or planes. A family of small molecule warheads defined by a combination of 4-fluoro-3-nitrobenzoic acid (BB1) and 3-(2-(tert-butoxy)ethyl) aniline (BB3) was identified through a highly populated line in the cube view plot (Fig. 4b). The most enriched warhead from this line is defined by a trisynthon combination of 4-fluoro-3-nitrobenzoic acid (BB1), 4-formylbenzoic acid (BB2), and 3-(2-(tert-butoxy)ethyl)aniline (BB3) and shown significant higher copy numbers than other warheads on the line. This most enriched warhead was chosen for hit confirmation by off-DNA synthesis which yielded the small molecule, GSK761 (Fig. 4b, c). More details about the development of a selective inhibitor of SP140 (GSK761) are added in Additional file 16.

The text describing the discovery and the synthesis of different other compounds (GSK064, GSK675, and GSK306) is added as Additional file 16.

### Fluorescence polarization (FP) binding affinity studies

6HisFLAGTEVSP140 (687-867) was serially diluted in the presence of 3 nM GSK064 in assay buffer (50 mM HEPES, pH 7.5, 150 mM NaCl, 0.05% Pluronic F-127, 1 mM DTT) in a total assay volume of 10 μl. Following incubation for 60 min, FP was measured on a Perkin Elmer Envision multi-mode plate reader, by exciting the Alexa647 fluorophore of GSK064 at a wavelength of 620 nm and then measuring emission at 688 nm in both parallel and perpendicular planes. The FP measurement, expressed as milliP (mP), was then calculated using the following equation;

$$mP=\frac{\left(\mathrm{parallel}\ \mathrm{fluorescence}\ \mathrm{intensity}\right)-\left(\mathrm{perpendicular}\ \mathrm{fluorescence}\ \mathrm{intensity}\right)}{\left(\mathrm{parallel}\ \mathrm{fluorescence}\ \mathrm{intensity}\right)+\left(\mathrm{perpendicular}\ \mathrm{fluorescence}\ \mathrm{intensity}\right)}$$

These data were then used to calculate an apparent *K*_d_ value by application of the following Langmuir-Hill equation; *AB* = (*A*0 + *B*)/(*K*d + *B*) where *AB* is the concentration of the bound complex, *A*_0_ is the total amount of one binding molecule added, *B* is the free concentration of the second binding molecule, and *K*_d_ is the dissociation complex. For IC_50_ determination, GSK761 was serially diluted in DMSO (1% final assay concentration) and tested in the presence of 50 nM SP140 and 3 nM GSK064 in the same assay buffer and volume as above. Reactions were incubated for 60 min, and IC_50_ values calculated using a four-parameter logistic equation; $y=x/\left[1+\left(\frac{I}{IC50}\right)n\mathrm{H}+D\right]$ where *y* is the mP signal in the presence of the inhibitor at concentration I, *x* is the mP signal without the inhibitor, IC_50_ is the concentration of the inhibitor that gives 50% inhibition, *nH* is the Hill coefficient, and *D* is the assay background.

### Pull-down of endogenous SP140 and Halotag-transfected SP140 with GSK761

HuT78 cells were stimulated with 100 ng/mL anti-CD3 antibody (in-house reagent) and 3 mg/mL anti-CD28 antibody (in-house reagent) for 96 h. Nuclear extract from HuT78 cells (endogenous SP140) was prepared following the manufacturer’s protocol (Active Motif nuclear extract and co-IP kit). Protein concentration was determined by Bradford Assay (Pierce). Nuclear extracts were incubated with GSK675 (biotinylated GSK761) prebound to streptavidin Dynabeads (Thermo Fisher). Dynabeads were incubated with 2X SDS reducing buffer (Invitrogen) and the eluted proteins resolved on a 4–12% Bis-Tris SDS-PAGE (Invitrogen) with MagicMark (Invitrogen) and SeeBlue 2 (Invitrogen) protein ladders. The gel was subjected to Western blotting (Invitrogen) and incubated with Rabbit anti-SP140 antibody (HPA-006162; Sigma) followed by anti-rabbit IgG HRP (A4914; Sigma). The Western blot was developed with Super Signal West femto kit (Thermo Fisher) and imaged on Carestream chemilluminescence imager (Kodak). A chimeric gene encoding Halo-SP140 was synthesized using overlapping oligonucleotides and cloned in an expression vector pCDNA3.1. HEK293 cells were transfected with an expression vector using Lipofectamine and incubated for 24 h. The transfected cells were labelled with HaloTag TMR ligand following the manufacturer’s protocol (HaloTag® TMR Ligand Promega). Nuclear extract from transfected HEK293 cells (Halo-SP140) was prepared, and SP140 immunoprecipitation was carried out as described above. Eluted proteins were resolved on a 4–12% Bis-Tris SDS-PAGE gel (Invitrogen) with SeeBlue 2 (Invitrogen) protein ladder. Halo-SP140 was visualized using Versa Doc scanner and 520LP UV Transilluminator. The original images of the full-length blots from which Fig. 4g was derived are added in Additional file 1: Fig. S11.

### BROMOscan® bromodomain profiling

BROMOscan® bromodomain profiling was provided by Eurofins DiscoverX Corp (Fremont, CA, USA, http://www.discoverx.com). Determination of the *K*_d_ between test compounds and DNA-tagged bromodomains was achieved through binding competition against a proprietary reference immobilized ligand.

### Cell penetration assessment assay of GSK761

Intracellular GSK761 compound concentration was measured by RapidFire Mass Spectrometry utilizing the methodology described by [33].

### Cell viability and cytotoxicity assays

“M1” macrophages were generated in vitro from human primary CD14^+^ monocytes as described above. “M1” macrophages were plated into an opaque-walled 96-well plate at 10 × 10^5^ cells per well and incubated with a concentration gradient of GSK761 (0.04–1.11 μM) for 1 h (0.1% DMSO was used as control). The cells were left unstimulated or stimulated with 100 ng/mL LPS for 24 h. Cell viability was assessed using CellTiter-Glo® Luminescent Cell Viability Assay kit (Promega) according to the manufacturer’s protocol. This assay quantifies ATP, an indicator of metabolically active cells. An equal volume of freshly prepared CellTiter-Glo® reagent was added to each well, the plate was shaken for 10 min at RT, and luminescent signals were recorded using a plate reader (SpectraMax M5). The index of cellular viability was calculated as the fold change of luminescence with respect to untreated control cells.

Cell viability was also determined using Muse® Count & Viability Kit (Luminex Corp) according to the manufacturer’s protocol and measured on the Guava® Muse® Cell Analyzer (Luminex Corp).

### Histone H3 peptide displacement

Various H3 peptides (Anaspec library (https://www.anaspec.com/)) were serially diluted in DMSO (1% final assay concentration) and tested in the presence of 10 nM GSK306 (FAM-labelled version of GSK761) and 80 nM 6HisFLAGTEVSP140 (687-867) (2x apparent *K*_d_ for this ligand) in 50 mM HEPES (Sigma-Aldrich), pH 7.5, 50 mM NaCl (Sigma-Aldrich), 1 mM CHAPS (Sigma-Aldrich), and 1 mM DTT (Sigma-Aldrich) in a total volume of 10 μl. Reactions were incubated for 30 min, and FP was measured on a Perkin Elmer Envision multi-mode plate reader (PerkinElmer), by exciting the FAM fluorophore of GSK306 at a wavelength of 485 nm and then measuring emission at 535 nm in both parallel and perpendicular planes. The FP measurement, expressed as milliP (mP), was then calculated as described previously, and normalized to free and bound ligand controls to determine % response. IC_50_ values were calculated using the described four-parameter logistic equation.

### Modified histone peptide array with SP140

Modified histone peptide arrays (Active Motif) were used to screen the interaction of SP140 PHD-Brd with 59 acetylation, methylation, phosphorylation, and citrullination modifications on the N-terminal tails of histones H2A, H2B, H3, and H4. Each peptide array contains 384 unique histone modification combinations in duplicate. In brief, the array slides were incubated with 3% BSA (Sigma) to block non-specific binding sites, followed by 10 mg/mL of 6His-Flag-Tev-SP140-PHD-Brd (687-867) protein or with no protein (negative control). The slides were then washed and incubated with α-6His Tag antibody (ab9108, Abcam) and α-rabbit IgG HRP antibody (A4914, Sigma) and stained with Super signal West femto kit (Thermo Scientific) according to the manufacturer’s instructions. The data were processed on Fujifilm LAS-3000 imager. All positive and negative signals and the corresponding peptide location can be tracked in Additional file 15: Table S15.

### SP140/histone nano-bioluminescence resonance energy transfer™ (NanoBRET™) assay testing

The NanoBRET™ System is a proximity-based assay that can detect protein interactions by measuring energy transfer from a bioluminescent protein donor NanoLuc® (NL) to a fluorescent protein acceptor HaloTag® (HT). Briefly, SP140-NL and Hitone3.3-HT DNA were transfected into HEK293 cells using the following ratios: 1:1, 1:10, and 1:100, respectively. Signal window was determined by relative NL/HT-fused protein expression. Minus HT controls were used as the baseline in this experiment for each condition. The data is presented as NanoBRET response (mBU) which is dependent on the presence of the HT Ligand, and by microscopic imaging of NL/HT-fused protein signal. For more information about NanoBRET assay protocol, check the following: https://www.promega.com/-/media/files/resources/protocols/technical-manuals/101/nanobret-proteinprotein-interaction-system-protocol.pdf?la=en.

### Flow cytometry (FACS)

Primary human CD14^+^ monocytes were positively selected from PBMCs using CD14 Microbeads according to the manufacturer’s instructions (Miltenyi Biotec) and differentiated for 3 days with 20 ng/mL M-CSF. The monocytes were then washed with PBS and treated with 0.1% DMSO or 0.04 μM GSK761. After 1 h, 100 ng/mL IFN-γ (R&D systems) was added to the cells to generate “M1” macrophages. After 3 days of incubation, the cells were harvested and subsequently permeabilized using 1% Saponin (Sigma-Aldrich) for 10 min on ice. Cells were then stained using the conjugated antibodies; PerCP-Cy5 mouse anti-human CD64 (dilution 1:50) (305023, Biolegend) and PE mouse anti-human CD206 (dilution 1:20) (2205525, Sony Biotechnology). The analysis was performed by flow cytometry (FACS) using the LSRFortessa and FACSCalibur (both BD Biosciences). FlowJo (BD LSRFortessa™ cell analyzer) was used for data analysis.

### Cytokine analysis

Cytokine expression in the supernatant of DMSO- or GSK761-treated “M1” macrophages (LPS-stimulated or unstimulated) were measured using electro-chemiluminescence assays (Meso Scale Discovery [MSD])-Human ProInflammatory 7-Plex Tissue Culture Kit (IFN-γ, IL-1β, IL-6, IL-8, IL-10, IL-12p70, TNF) according to the manufacturer’s protocols and analyzed on an MSD 1250 Sector Imager 2400 (Mesoscale). Supernatant IL-6, IL-8, and TNF from *SP140* siRNA or scrambled-treated “M1” macrophages (LPS-stimulated or unstimulated) were determined by sandwich enzyme-linked immunosorbent assay (ELISA; R&D systems) according to the manufacturer’s protocol.

### Customized qPCR array

Human “M1” macrophages were generated in vitro from human primary CD14^+^ monocytes as described above (from 5 independent donors). “M1” macrophages were treated either with 0.1% DMSO or 0.04 μM GSK761 for 1 h. The cells were then washed with PBS and stimulated with LPS for 4 h. Total RNA was isolated using RNeasy Mini Kit (Qiagen) and treated with DNaseI (Qiagen) according to the manufacturer’s instructions. RNA was reverse transcribed using the First-Strand Synthesis Kit (Qiagen) and loaded onto a customized RT [2] profiler array for selected 89 genes according to the manufacturer’s instructions (Qiagen) and run on QuantStudio 7 Flex (software v1.0). Qiagen’s online GeneGlobe Data Analysis Center (https://geneglobe.qiagen.com/us/analyze/) was used to determine the DEGs. The data was presented as a scatter plot. All data were normalized to the geometric mean of two reference genes (*RPL37A* and *ACTB*). The list of genes included in this experiment was selected from DEG in *SP140* silenced “M1” macrophages in [10] and from our MSD, qPCR, and microarray datasets of *SP140* silenced “M1” macrophages.

### Genome-wide expression profiling (RNA sequencing (RNA-seq))

“M1” macrophages were generated in vitro from human primary CD14^+^ monocytes as described above (from 3 independent donors). “M1” macrophages were incubated for 1 h with either 0.1% DMSO or 0.04 μM GSK761. “M1” macrophages were then kept unstimulated or stimulated with 100 ng/mL LPS (*E. coli* 0111:B4; Sigma) for 4 or 8 h. Total RNA was isolated from macrophages using the RNAeasy mini kit (Qiagen) and transcribed into cDNA by qScript cDNA SuperMix (Quanta Biosciences) according to the manufacturer’s instructions. Sequencing of the cDNA was performed on the Illumina HiSeq4000 to a depth of 35M reads at the Amsterdam UMC Core Facility Genomics. Quality control of the reads was performed with FastQC (v0.11.8) and summarization through MultiQC (v1.0). Raw reads were aligned to the human genome (GRCh38) using STAR (v2.7.0) and annotated using the Ensembl v95 annotation. Post-alignment processing was performed through SAMtools (v1.9), after which reads were counted using the featureCounts application in the Subread package (v1.6.3). Differential expression (DE) analysis was performed using DESeq2 (v1.24.0) in the R statistical environment (v3.6.3), in R2 Genomics Analysis and Visualization Platform-UMC (r2.amc.nl) and ShinyGO v0.60 [72].

### Chromatin immunoprecipitation (ChIP)

“M1” macrophages were generated in vitro from human primary CD14^+^ monocytes as described above (10^7^ cells were used for each condition). “M1” macrophages were either incubated for 1 h with 0.1% DMSO or with 0.04 μM GSK761 and left unstimulated or simulated with 100 ng/mL LPS for 1 or 4 h. The cells were cross-linked with 1% formaldehyde for 10 min at RT and quenched with 2.5 M glycine (Diagenode) for 5 min at RT. The ChIP assay was performed using the iDeal ChIP kit for Transcription Factors (Diagenode) and sonication was performed using the Picoruptor^TM^ (Diagenode) according to the manufacturer’s protocols. Chromatin shearing was verified by migration on a 1% agarose gel (E-Gel, Thermo Fisher) and visualized using E-Gel imager (Thermo Fisher). Immunoprecipitation was performed with a polyclonal SP140 antibody (H00011262-M07, Abnova). DNA was purified using IPure kit (Diagenode) according to the manufacturer’s protocol. For validation, quantitative real-time ChIP-qPCR was performed on DNA isolated from input (unprecipitated) chromatin and SP140 ChIP DNA with primer pairs specific for the TSS of *TNF* and *IL6* genes. For detailed PCR primer sequences, please see Additional file 4: Table S3. Quantitative PCR was performed using SYBR Green (Applied Biosystems) and StepOnePLus (Applied Biosystems). Results were quantitated using the delta–delta CT (ΔΔCT) method. Libraries of input DNA and ChIP DNA were prepared from gel-purified >300–base pair DNA.

### Global profiling of chromatin binding sites

The DNA was used to generate sequencing libraries according to the manufacturer’s procedure (Life Technologies). The DNA was end polished and dA tailed, and adaptors with barcodes were ligated. The fragments were amplified (eight cycles) and quantified with a Bioanalyzer (Agilent). Libraries were sequenced using the HiSeq PE cluster kit v4 (Illumina) with the HiSeq 2500 platform (Illumina), resulting in 125-bp reads. FastQ files were adapter clipped and quality trimmed using BBDukF (java -cp BBTools.jar jgi.BBDukF -Xmx1g in=fastqbef.fastq.gz out=fastq.fastq minlen=25 qtrim=rl trimq=10 ktrim=r k=25 mink=11 hdist=1 ref=adapters.fa). Subsequently, the reads were mapped to NCBI37/HG19 using Bowtie (bowtie2 -p 8 -x hg19 fastq.fastq > sample.sam) and sorted and converted into bam files using samtools. Peaks were called using MACS2, with the reads being extended to 200 bp (callpeak --tempdir /data/tmp -g hs -B -t sample.bam -c control.bam -f BAM -n result/ --nomodel --extsize 149) and duplicates removed. BDG files were binned in 25-bp regions and loaded in the R2 platform (r2.amc.nl) for subsequent analyses and visualization. For motif analyses, Summits obtained from the MACS2 analyses were extended by 250 bases and subsequently analyzed on a repeat masked hg19 genome with HOMER to identify over-represented sequences (both known as well as de novo). The data was uploaded and analyzed in R2 Genomics Analysis and Visualization Platform-UMC (r2.amc.nl).

### SP140 inhibition in isolated human mucosal macrophages

Colon tissues were obtained during surgery procedures from patients with CD. Patients’ characteristics are added as Additional file 14: Table S14. The mucosa was stripped and dissociated in GentleMACS tubes in digestion medium (DM; complete medium (RPMI 1640 with PGA/L-glutamine/10% FCS) with 1 mg/mL Collagenase D (Roche), 1 mg/mL soybean Trypsin inhibitor (Sigma), 50 μg/mL DNase I (Roche)), and then mechanically dissociate on GentleMACS using program B01. The mucosa was then incubated in DM for 1 h at 37 °C while shaking. During digestion, the dissociation was repeated two times. The tissue suspension was passed through a cell strainer (200–300 um) and centrifuged at 1500 rpm for 10 min. The dissociated cells were resuspended in cold MACS Buffer, and macrophages were isolated using CD14 MicroBeads according to the manufacturer’s instructions (MiltenyiBiotec). The macrophages were incubated with either 0.1% DMSO or 0.04 μM GSK761 for 4 h. RNA was extracted as described above, and gene expression of *IL6*, *TNF*, *IL10*, and *CD64* was measured by qPCR. All data were normalized to the reference gene *ACTB.*

### Statistics analysis

Statistical analysis was performed with GraphPad Prism v8.0.2.263 (GraphPad Software Inc.). For group analysis, data were subjected to one-way ANOVA or Student’s *t* test. The two-tailed level of significance was set at *p* ≤ 0.05 (*), 0.01 (**), 0.001 (***), or 0.0001 (****) for group differences. Data is shown as mean ± SEM. The figures were prepared using Inkscape 0.92.4.

## Supplementary Information

**Additional file 1: Supplementary figures 1-11: Supplementary figure 1.** SP140 expression is associated with inflammatory diseases. (a) SP140 protein harbors three functional domains: the epigenetic readers bromodomain (Brd) and a plant homeodomain (PHD) finger that dock histone post-translational modifications (acetylation and methylation marks, respectively) and SAND DNA-binding protein domain that docks DNA. In addition, SP140 also contains nuclear localization signal (NLS) and homogeneously-staining region (HSR). (b) Immunohistochemistry of SP140 in ulcerative colitis (colon), appendicitis (appendix), sarcoidosis (lung), psoriatic arthritis (synovium), rheumatoid arthritis (synovium), Hashimoto’s thyroiditis (thyroid) and Sjogren’s syndrome (cervical cyst). SP140 is illustrated by peroxide staining. **Supplementary figure 2.** Cell clustering analysis in ileal tissue. Publicly available single-cell RNA sequencing (Martin et al) was used to illustrate *SP140* expression in intestinal (ileum) macrophages in inflamed (*n*=11) and uninflamed (*n*=11) tissue CD patients. (a and b) UMAP annotated by the 22 clusters as identified on the basis of the top 2000 most variable genes and the top 15 principal components alongside the marker genes used to identify the various cell types. (c) Visual illustration of the expression of various monocyte, macrophage and dendritic cell markers. Darker blue represents more reads per cell. (d) Comparative abundance analyses of the different cell types when comparing inflamed with uninflamed. Graphs are ranked by *p*-values, which were calculated through the Wald test as implemented in DESeq2. (e) Subsequent clustering analyses of the MNPs in cluster 6 were performed using the top 2000 most variable genes and the top 5 principal components yielding 10 subclusters. (f) Visual illustration of the estimated trajectory of cell development along the monocyte-to-macrophage/DC axis. **Supplementary figure 3.** Inflammatory stimulus induces SP140 expression. (a,b) Relative gene expression of *CD64*, *CCL5*, *CD206* and *CCL22* in “M0”, “M1” and “M2” macrophages derived from (a) human primary CD14^+^ monocytes or (b) from THP-1 cells, *n*=4. (c) Relative gene expression of *SP140* in “M0”, “M1” and “M2” macrophages derived from THP-1 cells, *n*=5. (d) Immunofluorescence staining of SP140 in “M1” and “M2” macrophages derived from THP-1 cells. DAPI staining nucleus in blue and SP140 speckles in red. Scale bar: 3μm. (e) Relative gene expression of *SP140*, *CCL5* and *CD206* after 24h of 100 ng/mL LPS-stimulated or unstimulated “M0”, “M1” and “M2” macrophages derived from human primary CD14^+^ monocytes, *n*=4. Relative gene expression was measured by qPCR. Statistical significance is indicated as follows: *P < 0.05, **P < 0.01, ***P < 0.001, ****P < 0.0001. **Supplementary figure 4.** Expression of BCPs in inflammatory macrophage subsets. (a) Relative gene expression (qPCR) of 12 BCPs; *SP100*, *SP110*, *SP140L*, *BRD2*, *BRD3*, *BRD4*, *BRD9*, *PCAF*, *EP300*, *CRBBP*, *BAZ2A* and *BAZ2B* in “M0”, “M1” and “M2” macrophages derived from human primary CD14^+^ monocytes, *n*= 4 to 8. (b) Relative gene expression of *SP110* and *SP100* in “M1” macrophages, transfected with siRNA against *SP140* (gray bars) or a scrambled control siRNA (black bars), and then stimulated or not with LPS (100 ng/mL for 4h). (c) Immunofluorescence staining of SP140 in “M1” macrophages, transfected with siRNA against *SP140* or a scrambled control siRNA, and then stimulated with LPS (100 ng/mL for 24h). SP140 speckles (red dots) were imaged and SP140 nuclear bodies were counted. DAPI (blue) was used to stain the nucleus. Scale bar: 3μm. **Supplementary figure 5.** GSK761 lowers the expression of the M1 macrophage polarization marker *TNF* and supports the anti-inflammatory effect of infliximab. (a) ATP luminescence measurement within “M1” macrophages treated with an increasing concentration of GSK761 (0.01, 0.04, 0.12, 0.37. 1.11 μM) or 1% DMSO in presence or absence of 100 ng/mL of LPS for 24h, *n*=6, statistical significance is indicated as follows: *P < 0.05, **P < 0.01. The Y axis indicates the fold change in detected ATP luminescence (relative to unstimulated DMSO control). (b) “M1” macrophages were pretreated with 0.1% DMSO or with GSK761 (0.04 μM, 0.12 μM or 0.37 μM), and then stimulated with 100 ng/mL for 24h. The cells were stained with Muse® Count & Viability Reagent, and then analyzed on the Muse® Cell Analyzer. The data shown as viability vs nucleated cells and presented as % viability. (c) Gating strategy (belongs to Fig. 1.b and d) (d) Primary human CD14^+^ monocytes were differentiated with 20 ng/mL M-CSF for 3 days. The cells were then washed with PBS and treated with 0.1% DMSO or 0.04 μM GSK761 for 1h prior to 3 days polarization to “M1” phenotype with 100 ng/mL INF-γ (DMSO and GSK761 were not washed and kept in the culture during the 3 days of polarization). *TNF* gene expression was measured by qPCR, *n*=3. (e) “M1” macrophages were treated with only media, 0.04 μM GSK761, 0.1 μg/mL Infliximab (anti-TNF antibody) or with both 0.04 μM GSK761 + 0.1 μg/mL Infliximab for one hour prior of LPS stimulation (100 ng/mL) for 24h. TNF protein release in the supernatant was measured with ELISA. **Supplementary figure 6.** SP140 protein binds to histone H3. (a) Modified histone peptide arrays screening the interaction of SP140-PHD-Brd (687-867) with 59 acetylation, methylation, phosphorylation and citrullination modifications on the N-terminal tails of histones peptides; H2A, H2B, H3 and H4. The experiment was performed in duplicate. (b) IC50 (0.36 +/- 0.03 μM) for the displacement of GSK761 from SP140 by H3 peptide (1-21, ARTKQTARKSTGGKAPRKQLA). (c) IC50 for the displacement of GSK761 by H3 peptide (circles) compared to IC50s for H3 peptide tri-methylated at position K4 (1-21, H3K4(3Me)) (Squares) and H3 peptide tri-methylated at position K14 (1-21, H3K14(3Me)) (10 +/- 1 and 0.28 + 0.03 μM, respectively). (c) IC50s for the displacement of a range of modified H3 and truncated peptides. Open circles; Biotinylated (B) H3 peptide (1-21, IC50 = 0.51 μM), open squares; Biotinylated H3K9(Ac) (1-21, IC50 = 2.22 μM), open triangles up; Biotinylated H3K4(3Me)K9(Ac) (1-21, IC50 = 11.32 μM), open triangles down; Biotinylated H3K4(3Me) (1-21, IC50 = 17.66 μM), open diamonds; biotinylated H3K14(Ac) (1-21, IC50= 0.43 μM), open octagon; H3K4(3Me) (1-10, IC50 = 12.47 μM), plus symbol; H3K4(3Me) (1-21, IC50 = 6.10 μM), star symbol; biotinylated H3(+YCK ) (1-18 +YCK, IC50 = 0.27 μM), solid circle; H3K4(Ac)K14(3Me) (1-21, IC50 = 13.10 μM), solid square; H3K4(3Me)K14(Ac) (1-21 IC50 = 9.99 μM and solid triangle up; biotinylated H3 (1-9 IC50 = 2.83 μM). (d) Peptides tested and data not shown (due to no fit); Biotinylated H3K14(Ac) (9-20), Biotinylated H3 (15-40) and Biotinylated H3 (5-24). (e) SP140-NL and Histone3.3-HT DNA were transfected into HEK293 cells. NL/HT-fused protein green signal was imaged by microscopy (left) and NanoBRET response (mBU) was measured (right). **Supplementary figure 7.** GSK761 reduced SP140 binding to the DNA. (a) SP140 precipitation with anti-SP140 polyclonal antibody (Sigma, HPA006162) in whole cell lysate from HuT78 cells, SP140 transfected HeLa cells and untransfected HeLa cells. (b) Metagene created from normalized genome-wide average reads for SP140 binding at immune innate (red) and non-immune innate genes (blue), centered on TSS. (c) Roadmap comparing the DMSO versus GSK761 on the peaks that were called in both instances (In 1h LPS-stimulated “M1” macrophages). If the peaks in both conditions are overlapping, then we call them unaffected (a peak is found under both conditions) (right). If there was a peak in DMSO, but in the presence of GSK761, SP140 binding is reduced or absent, then this is described as ‘SP140 reduced’ (middle). Finally, if there was no peak called in DMSO samples, but a peak appears in the GSK761 condition, then SP140 binding is described as 'SP140 appears' (left). For the circles: All have been compared to the epigenome roadmap data for 1 particular tissue (primary monocytes from peripheral blood). This provides an annotation for every 200bp region in the genome and states its association in 15 different categories. The narrow outer circle reflects the proportion of the annotated genome in that particular tissue (primary monocytes from peripheral blood). The thicker inner circle reflects the proportions of the annotated genome where SP140 binding is seen (base pair with the highest signal in a peak) for the peaks as termed above. The numbers inside the circles reflect the number of SP140 enriched genes. (d) SP140 enrichment at H3K27ac in “M1” macrophages after 0h, 1h and 4h of 100 ng/mL LPS stimulation. **Supplementary figure 8.** GSK761 prevents SP140 enrichment at gene set involved in TNF signaling and antigen presentation. (a) A complete data set of hallmark pathway analysis for global DEGs in LPS-unstimulated or stimulated (4 or 8h) “M1” macrophages (pretreated with 0.1% DMSO or 0.04 μM GSK761). The direction and color of the arrow indicate the direction and size of the enrichment score, the size of the arrow is proportional to the –log_10_ (*p*-value), and non-transparent arrows represents significantly affected pathways, *n*=3. (b) An enrichment pathway analysis targeting TNF signaling for SP140 ChIP-seq (top) and RNA-seq (bottom) comparing 0.1% DMSO- with 0.04 μM GSK761 treated-“M1” macrophages at 0, 1 and 4h of 100 ng/mL LPS stimulation. (c) R2 TSS plot targeting TNF signaling pathway genes, comparing 0.1% DMSO with 0.04 μM GSK761 treated “M1” macrophages after 1h of 100 ng/mL LPS stimulation. The indicated genes in the graph present the most differentially bound genes (DBGs). (d) Heatmap of most DEGs that are involved in TNF signaling, comparing 0.1% DMSO with 0.04 μM GSK761 treated “M1” macrophages after 4h of 100 ng/mL LPS stimulation. (e) SP140 ChIP-seq genome browser view of *HLA-DMB*, *HLA-DMA*, *HLA-DQA*, *HLA-DPA1*, *HLA-DPB1*, *HLA-DPB2*, *HLA-C*, *HLA-B*, *HLA-F*, *HLA-A* and *BRD2*. Y axis represents the signal score of recovered sequences in 0.1% DMSO and 0.04 μM GSK761 treated macrophages after 0, 1 and 4h of 100 ng/mL LPS stimulation. **Supplementary figure 9.** Genes that are involved in innate immune response showed the strongest concordant differential SP140 binding and differential gene expression.bComparative analyses of the top 1000 differentially bound genes (DBGs) (peak signal resulted from comparing DMSO- vs GSK761-treated M1) with their gene expression (Wald statistic) (resulted from comparing DMSO- vs GSK761-treated M1). Comparative of gene expression at 8h LPS with ChIP at 1h LPS (top) and gene expression at 8h LPS with ChIP at 4h LPS (bottom). **Supplementary figure 10.** GSK761 reduces SP140 enrichment at several chemokine genes. (a) R2 TSS plot of chemokine-signaling genes, comparing 0.1% DMSO with 0.04 μM GSK761-treated “M1” macrophages after 1h of 100 ng/mL LPS stimulation. The indicated genes in the graph represent the most DBGs involved in chemokine activity/response. (b, c) SP140 ChIP-seq genome browser view of some of most SP140 differentially bound chemokines at different time points of LPS stimulation in “M1” macrophages.SP140-bound chemokines were highlighted. (d) Volcano plot of DEGs involved in chemokine signaling/genes after 1 h of 100 ng/mL LPS stimulation. **Supplementary figure 11.** The images of the full-length blots from which Fig. 4g was derived. Blue arrows indicate which ones were used to prepare the Fig. 4g.

**Additional file 2: Table S1**. Summary statistics for microarray data (*SP140* siRNA-“M1” macrophages vs scrambled siRNA-“M1” macrophages).

**Additional file 3: Table S2**. Summary statistics for microarray data (*SP140* siRNA-“M1” macrophages stimulated with 4h LPS (100 ng/mL) vs scrambled siRNA-“M1” with 4h LPS (100 ng/mL)).

**Additional file 4: Table S3**. Primer sequences used in the quantitative PCR analysis of the genes of interest. **Table S4**. GSK761 screen against human BCPs (Bromoscan assay) reveals no binding at *Kd* of ≤ 30000 nM (for most of tested BCPs) and at *Kd* of ≤ 21000 nM for PBRM1(5), indicating a high degree of specificity of GSK761 for SP140.

**Additional file 5: Table S5**. Counts and summary statistics for RNA-seq data (GSK761-pretreated “M1” macrophages vs DMSO-pretreated “M1” macrophages).

**Additional file 6: Table S6**. Counts and summary statistics for RNA-seq data (GSK761-pretreated “M1” macrophages stimulated with 4h LPS (100 ng/mL) vs DMSO-pretreated “M1” macrophages stimulated with 4h LPS (100 ng/mL)).

**Additional file 7: Table S7**. Counts and summary statistics for RNA-seq data (GSK761-pretreated “M1” macrophages stimulated with 8h LPS (100 ng/mL) vs DMSO-pretreated “M1” macrophages stimulated with 8h LPS (100 ng/mL)).

**Additional file 8: Table S8.** The normalized read count for ChIP-seq (DMSO-pretreated “M1” macrophages).

**Additional file 9: Table S9**. The normalized read count for ChIP-seq (DMSO-pretreated “M1” macrophages stimulated with 1h LPS (100 ng/mL)).

**Additional file 10: Table S10**. The normalized read count for ChIP-seq (DMSO-pretreated “M1” macrophages stimulated with 4h LPS (100 ng/mL)).

**Additional file 11: Table 11.** The normalized read count for ChIP-seq (GSK761-pretreated “M1” macrophages).

**Additional file 12: Table S12.** The normalized read count for ChIP-seq (GSK761-pretreated “M1” macrophages stimulated with 1h LPS (100 ng/mL)).

**Additional file 13: Table S13.** The normalized read count for ChIP-seq (GSK761-pretreated “M1” macrophages stimulated with 4h LPS (100 ng/mL)).

**Additional file 14: Table S14.** Characteristics Table of anti-TNF refractory CD patients.

**Additional file 15: Table S15.** Peptides location (Modified Histone Peptide Array).

**Additional file 16.** The discovery and the synthesis of GSK761, GSK064, GSK675 and GSK306.

## Data Availability

Microarray data have been deposited in the ArrayExpress database at EMBL-EBI (www.ebi.ac.uk/arrayexpress) under accession number E-MTAB-12011 [73]. Microarray data is also publicly available and can be explored at r2.amc.nl under the data set name “Exp SP140 siRNA in “M1” macrophages - deJonge - 12 - custom - ilmnht12v4.” The summary statistics for microarray data can be found in Additional file 2: Table S1 and Additional file 3: Table S2. Raw data of RNA-seq and ChIP-seq are available with controlled access for research purposes only and have been deposited at the European Genome-phenome Archive (EGA) (https://ega-archive.org), which is hosted by the EBI and the CRG, under accession number EGAS00001004460 [74]. Access to these data set can be requested from the corresponding author; Wouter de Jonge. The counts and the summary statistics for RNA-seq data can be found in Additional files 5, 6 and 7: Tables S5, S6 and S7. The normalized read count for ChIP-seq data can be found in Additional files 8, 9, 10, 11, 12 and 13: Tables S8, S9, S10, S11, S12 and S13. Raw sequencing reads of publicly available single-cell RNA sequencing (Martin et al.) [24] of inflamed and uninflamed ileal tissues are retrieved from the Gene Expression Omnibus under accession number GEO: GSE134809 [75].
