# Supplementary material for: Modulation of macrophage inflammatory function through selective inhibition of the epigenetic reader protein SP140
Source: BMC Biol. 2022 Aug 19;20:182. doi: 10.1186/s12915-022-01380-6 (PMC9392322; doi:10.1186/s12915-022-01380-6)

Supplementary figure 1

a

Functional domains in human SP140 protien

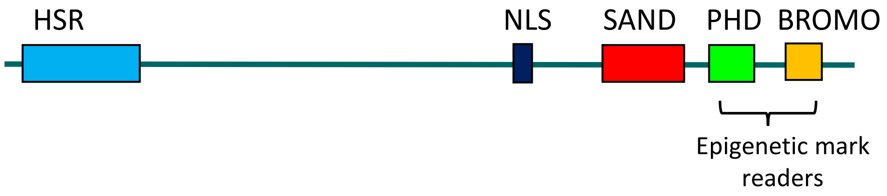

b

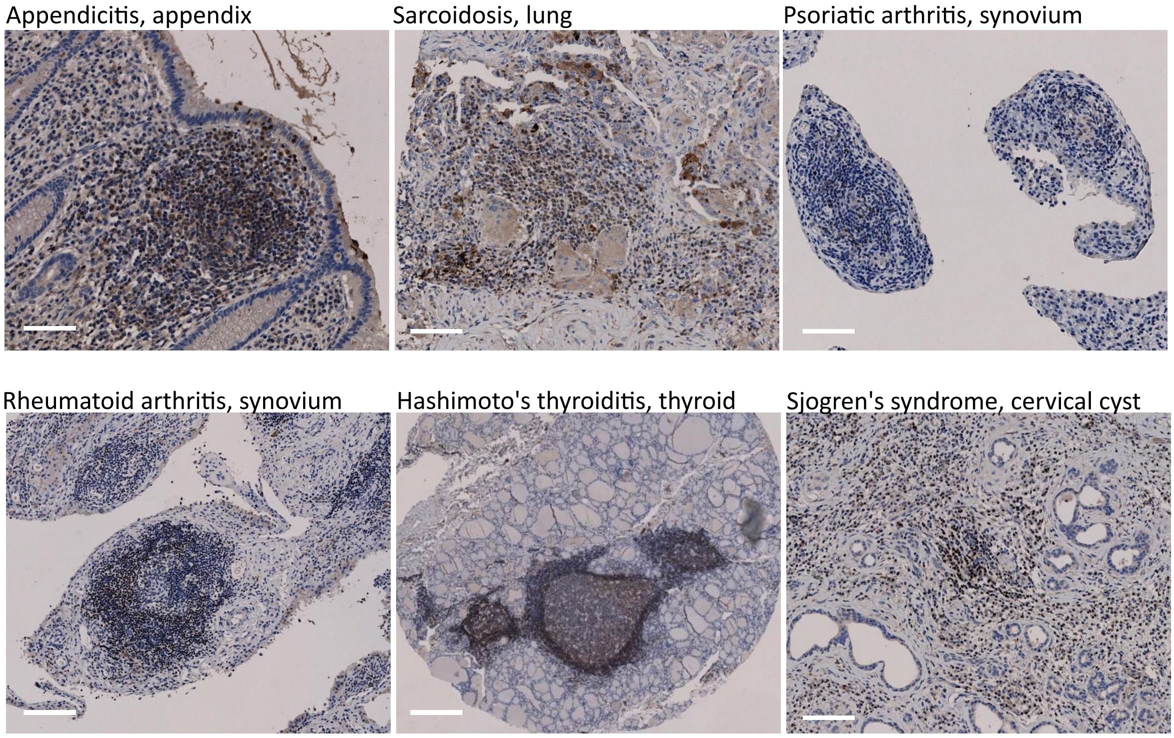

Supplementary figure 2

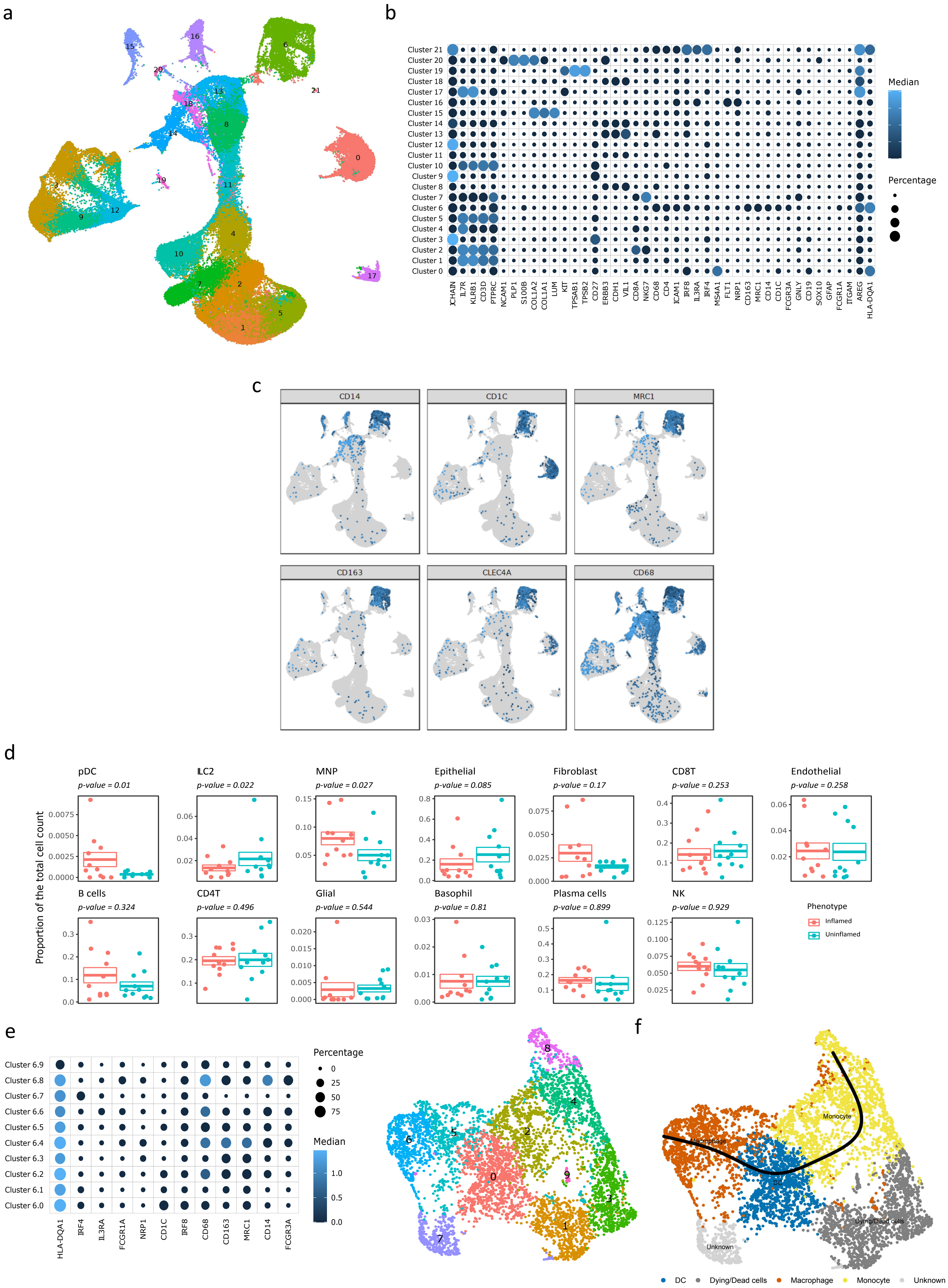

Supplementary figure 3

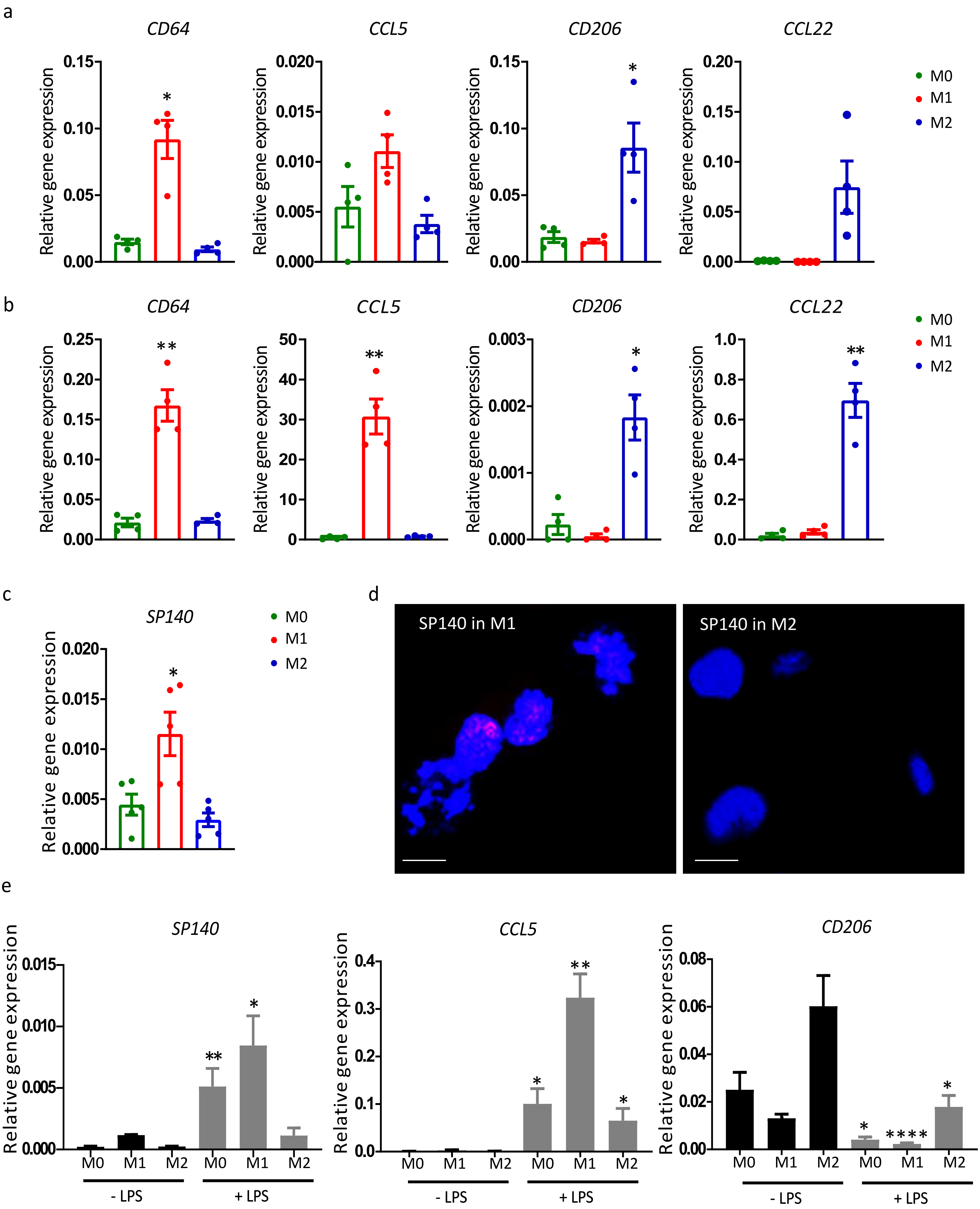

Supplementary figure 4

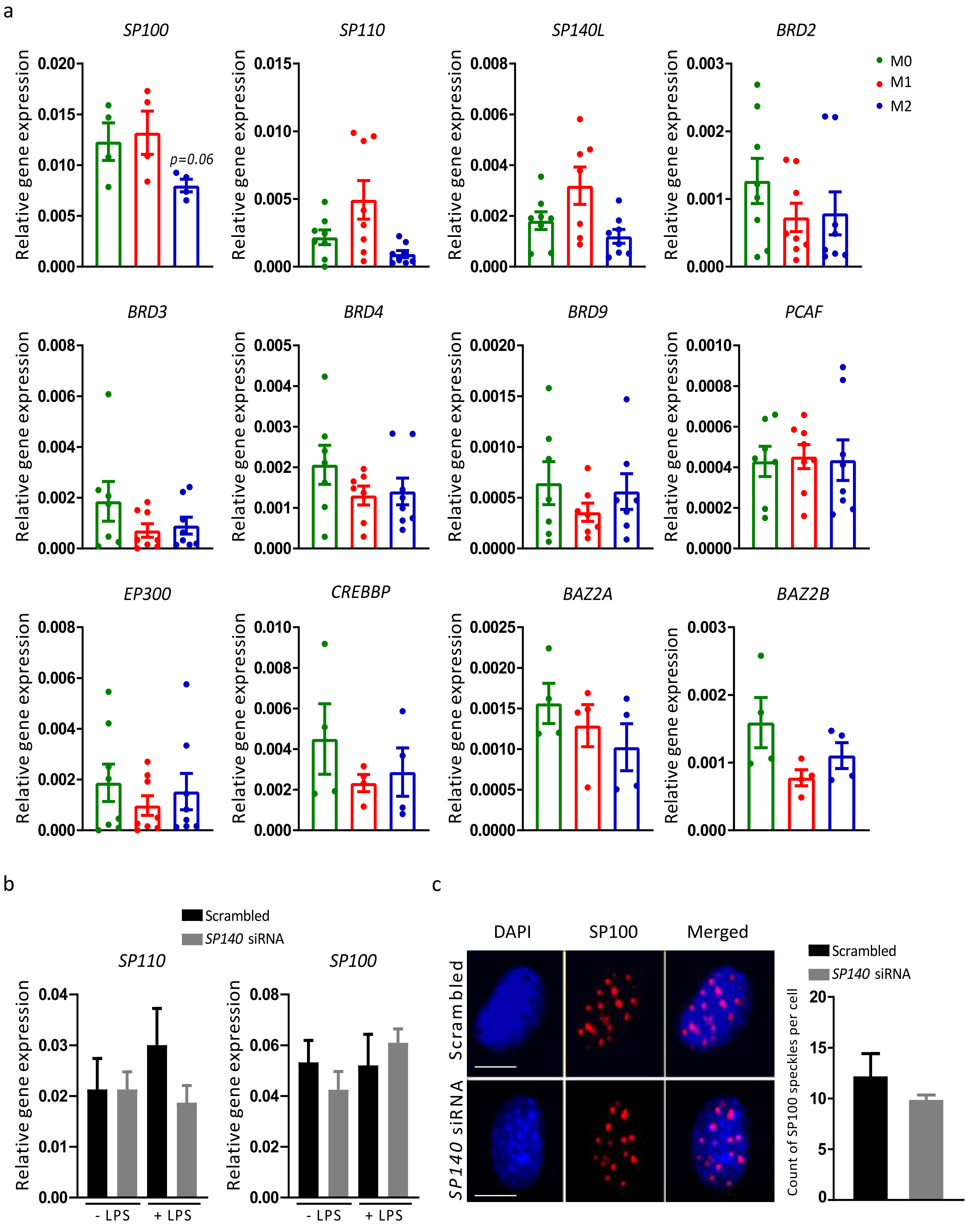

Supplementary figure 5

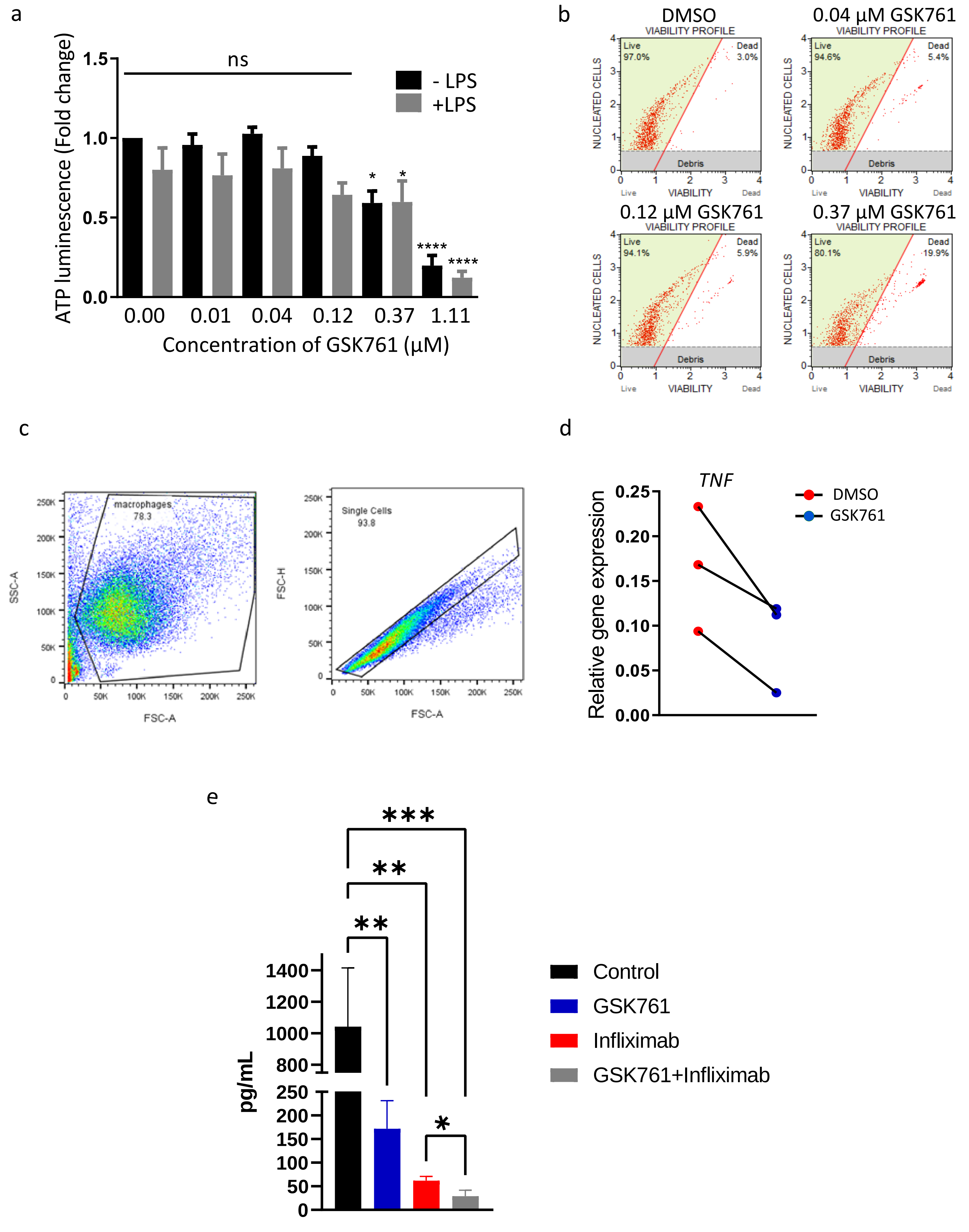

Supplementary figure 6

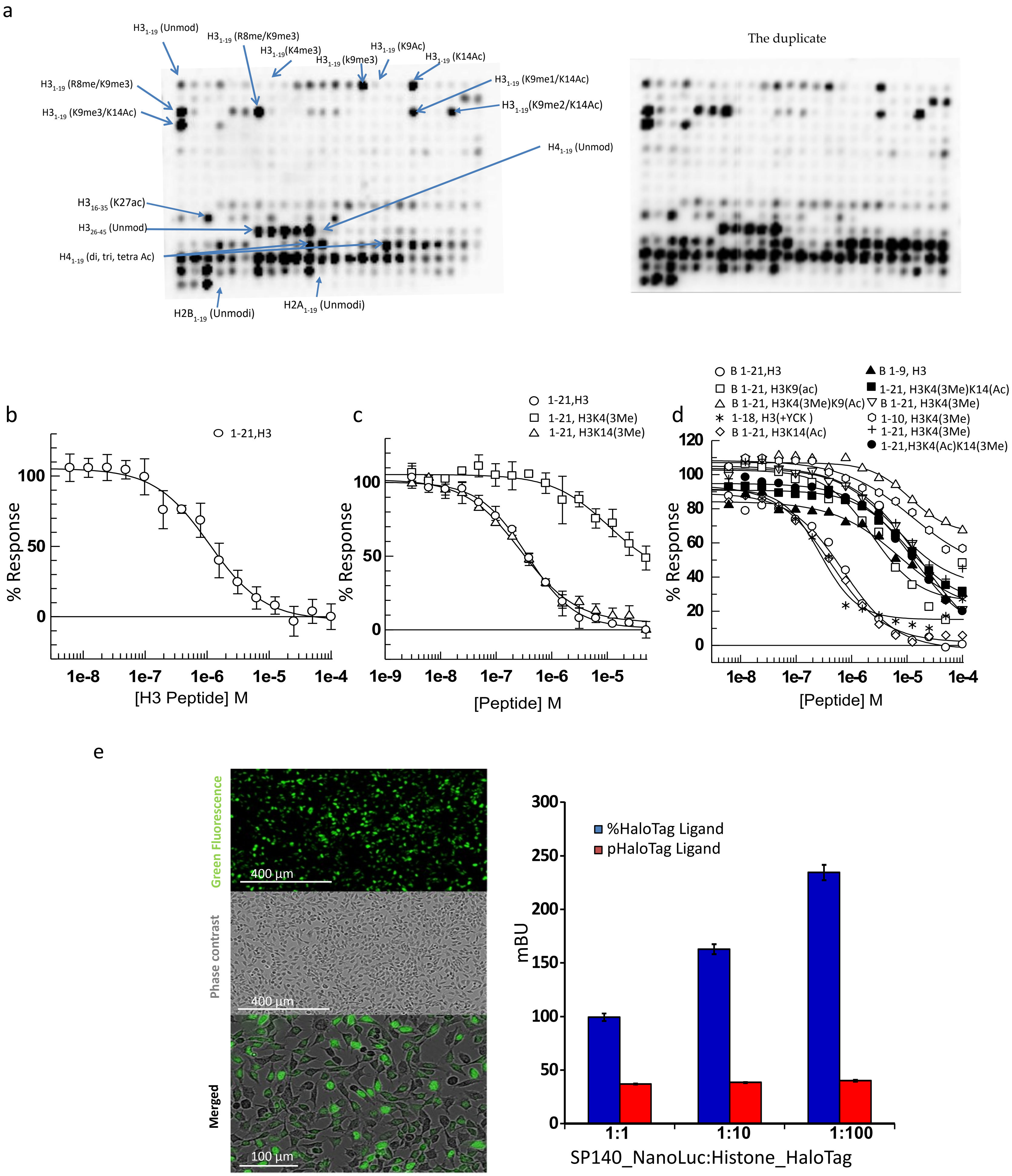

Supplementary figure 7

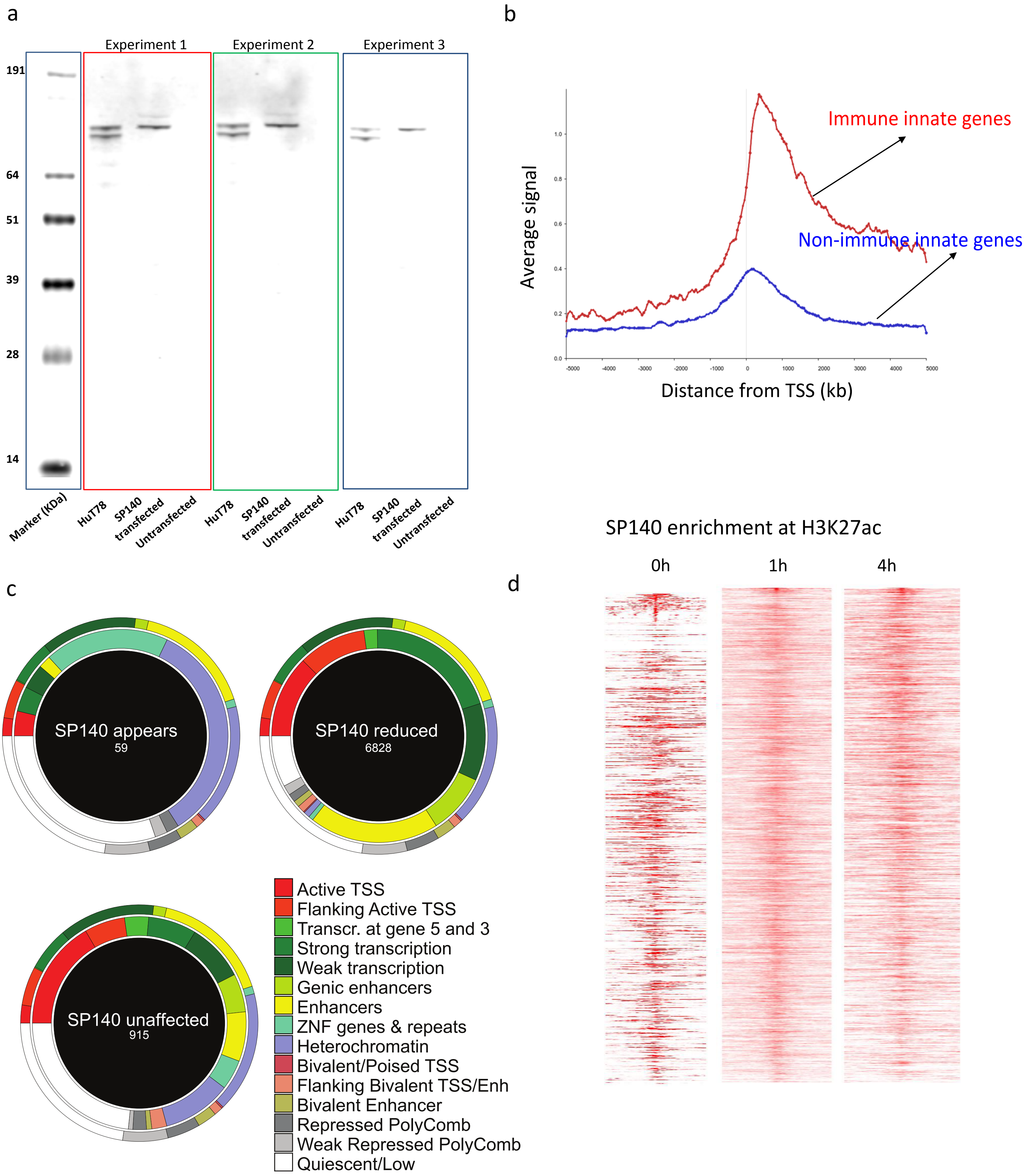

Supplementary figure 8

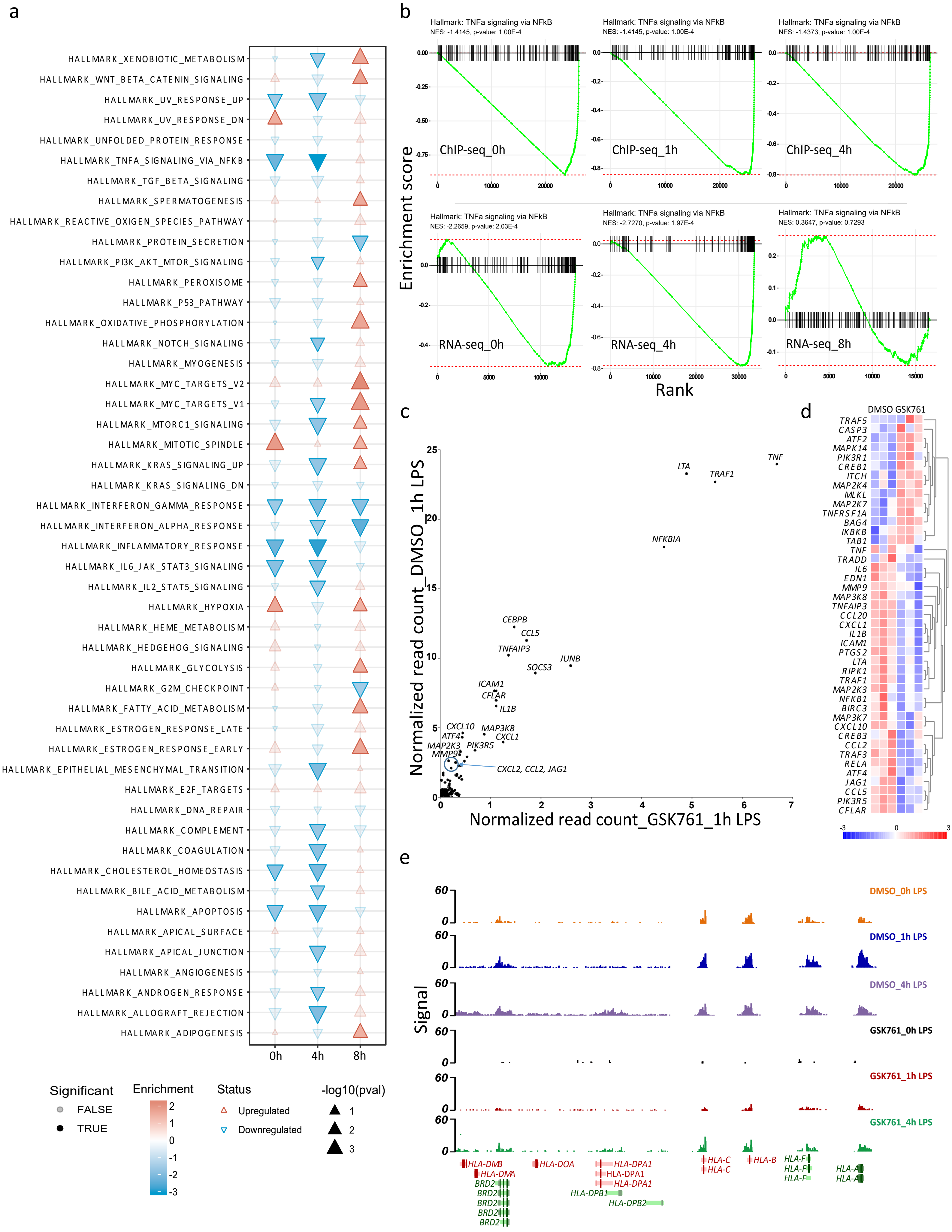

Supplementary figure 9

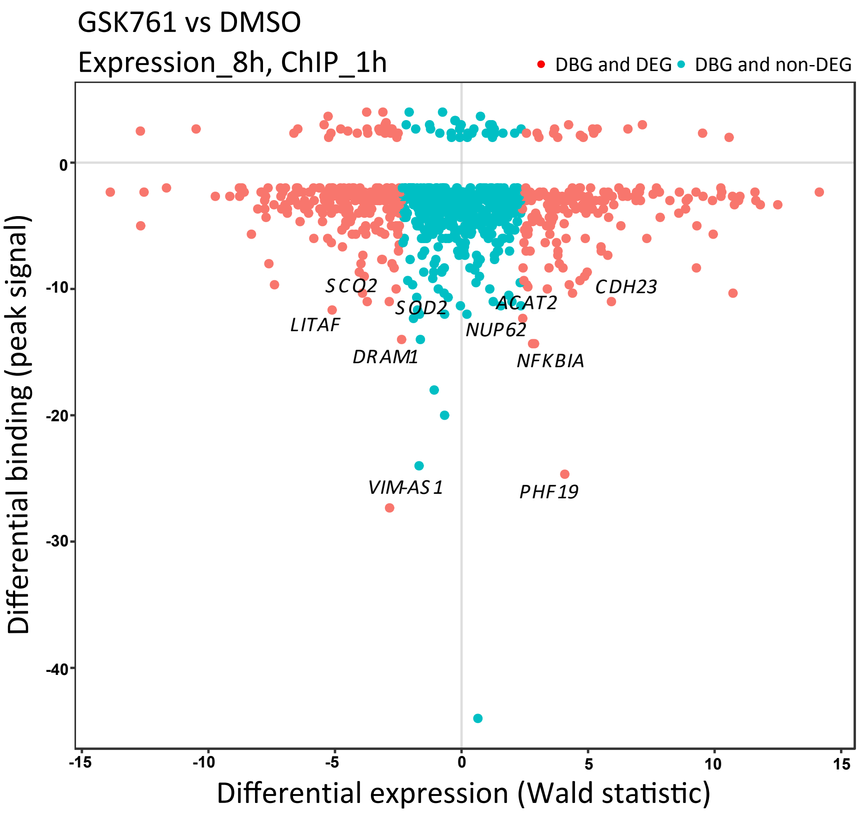

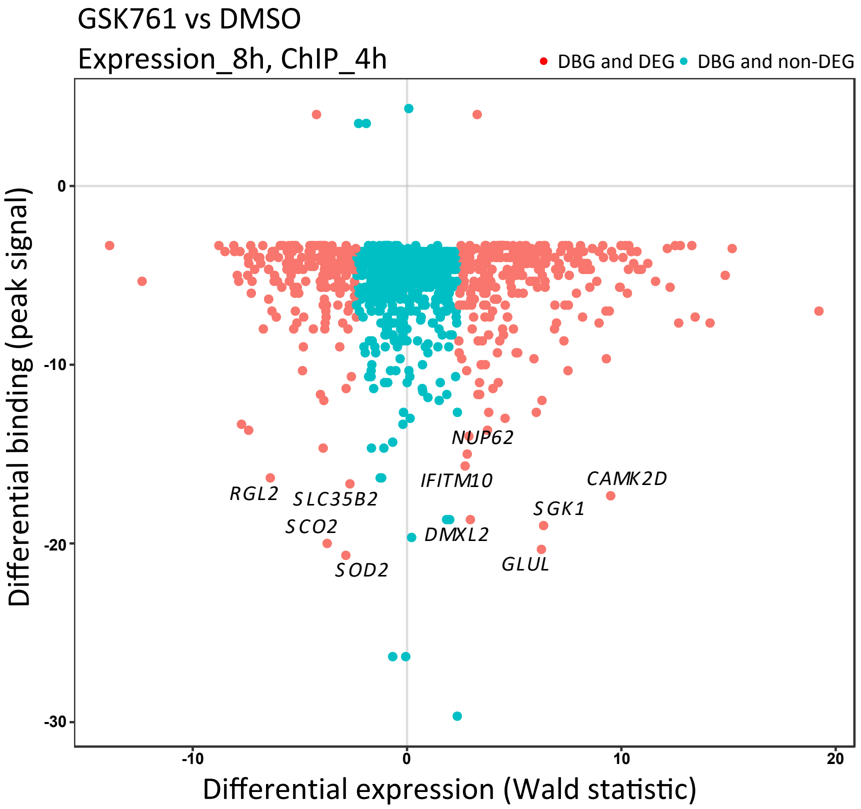

Supplementary figure 10

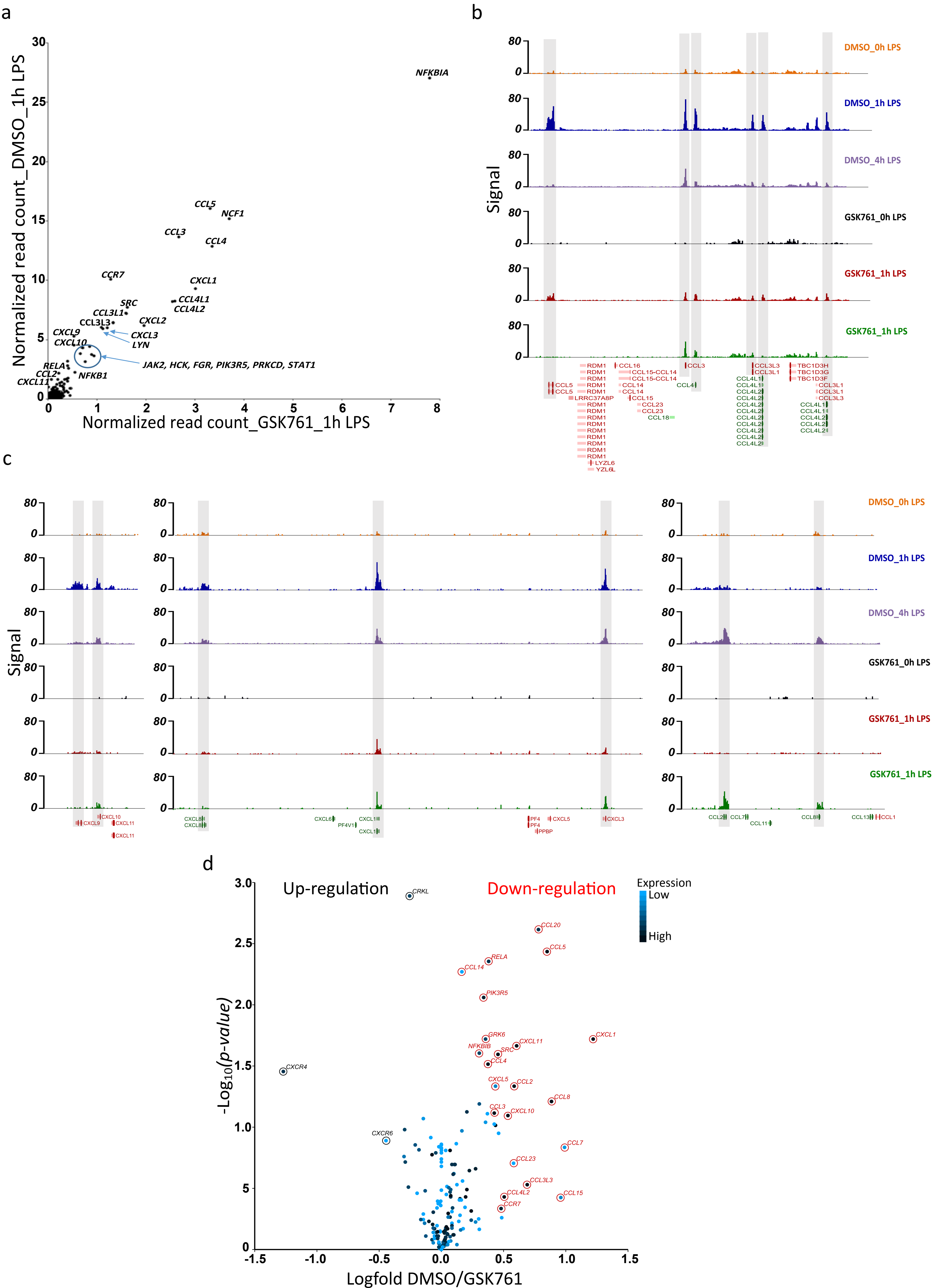

Supplementary figure 11

a

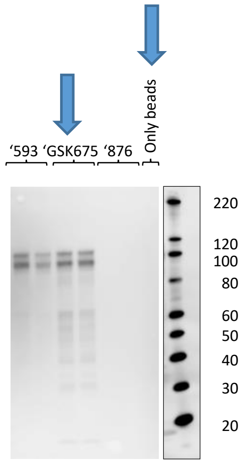

b

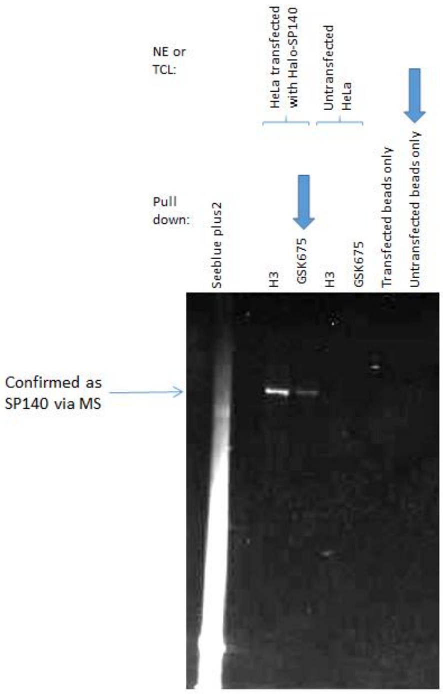

Supplement: Supplementary file 1 — Additional file 1: Supplementary figures 1-11: Supplementary figure 1. SP140 expression is associated with inflammatory diseases. (a) SP140 protein harbors three functional domains: the epigenetic readers bromodomain (Brd) and a plant homeodomain (PHD) finger that dock histone post-translational modifications (acetylation and methylation marks, respectively) and SAND DNA-binding protein domain that docks DNA. In addition, SP140 also contains nuclear localization signal (NLS) and homogeneously-staining region (HSR). (b) Immunohistochemistry of SP140 in ulcerative colitis (colon), appendicitis (appendix), sarcoidosis (lung), psoriatic arthritis (synovium), rheumatoid arthritis (synovium), Hashimoto’s thyroiditis (thyroid) and Sjogren’s syndrome (cervical cyst). SP140 is illustrated by peroxide staining. Supplementary figure 2. Cell clustering analysis in ileal tissue. Publicly available single-cell RNA sequencing (Martin et al) was used to illustrate SP140 expression in intestinal (ileum) macrophages in inflamed (n=11) and uninflamed (n=11) tissue CD patients. (a and b) UMAP annotated by the 22 clusters as identified on the basis of the top 2000 most variable genes and the top 15 principal components alongside the marker genes used to identify the various cell types. (c) Visual illustration of the expression of various monocyte, macrophage and dendritic cell markers. Darker blue represents more reads per cell. (d) Comparative abundance analyses of the different cell types when comparing inflamed with uninflamed. Graphs are ranked by p-values, which were calculated through the Wald test as implemented in DESeq2. (e) Subsequent clustering analyses of the MNPs in cluster 6 were performed using the top 2000 most variable genes and the top 5 principal components yielding 10 subclusters. (f) Visual illustration of the estimated trajectory of cell development along the monocyte-to-macrophage/DC axis. Supplementary figure 3. Inflammatory stimulus induces SP140 expression. (a, [file 12915_2022_1380_MOESM1_ESM.pdf]
