# Supplementary material for: Modulation of macrophage inflammatory function through selective inhibition of the epigenetic reader protein SP140
Source: BMC Biol. 2022 Aug 19;20:182. doi: 10.1186/s12915-022-01380-6 (PMC9392322; doi:10.1186/s12915-022-01380-6)
Supplement: Supplementary file 4 — Additional file 4: Table S3. Primer sequences used in the quantitative PCR analysis of the genes of interest. Table S4. GSK761 screen against human BCPs (Bromoscan assay) reveals no binding at Kd of ≤ 30000 nM (for most of tested BCPs) and at Kd of ≤ 21000 nM for PBRM1(5), indicating a high degree of specificity of GSK761 for SP140. [file 12915_2022_1380_MOESM4_ESM.pdf]

Supplemental Table 3. Primer sequences used in the quantitative PCR analysis of the genes of interest.

**Gene**	**Forward (5'-3')**	**Reverse (5'-3')**
SP140	AGGATGGTCGCAGAGATCCA	TGGCCTTGTTATTGCACTTGC
SP110	CAGCACACCTTCAGACAAGAA	TCTCTACCGTGGAGTTACAAGTT
SP100	TCCATGACAAATTGCCTCTCC	GAGATGGGGAACCCGAAGG
SP140L	GGGCTGAACGGAGGTGTTT	AGTGTCATAGACAAGTCCCTCAT
BRD2	GTGGTTCTCGGCGGTAAG	ACACCCCGGATTACATACCC
BRD3	TTGGCAAACCTCATCTCAAA	GATGTCCGGCTGATGTTCTC
BRD4	GATGGTGCTTCTTCTGCTCC	AGTCCAGCTCCTCTGACAGC
BRDT	TCCCTTGAACGTGGTACAGG	GCAGGAGTTGTTGTATCTGCT
BRD9	GCAATGACATACAATAGGCCAGA	GAGCTGCCTGTTTGCTCATCA
BAZ2A	ATGGAAATGGAGGCAAACGAC	GAGACCCGTTAGTGTAGAGGC
BAZ2B	ATGGAGTCTGGAGAACGGTTA	AATGTCCACATGGGTTGATTGT
EP300	TCTGGTAAGTCGTGCTCCAA	GCGGCCTAAACTCTCATCTC
PCAF	CGAATCGCCGTGAAGAAAGC	CTTGCAGGCGGAGTACACT
CREBBP	CAACCCCAAAAGAGCCAAACT	CCTCGTAGAAGCTCCGACAGT
CD64	ACCCCATACAGCTGGAAATC	TTATCCTTCCACGCATGACA
CD206	GGGTTGCTATCACTCTCTATGC	TTTCTTGTCTGTTGCCGTAGTT
CCL5	CCAGCAGTCGTCTTTGTCAC	CTCTGGGTTGGCACACACTT
CCL22	CGCGTGGTGAAACACTTCTA	GGATCGGCACAGATCTCCT
TNF	ATGTTGTAGCAAACCCTCAAGC	GGACCTGGGAGTAGATGAGGT
IL6	AGTGAGGAACAAGCCAGAGC	GTCAGGGGTGGTTATTGCAT
IL8	AAATTTGGGGTGGAAAGGTT	TCCTGATTTCTGCAGCTCTGT
ACTB	AATGTGGCCGAGGACTTTGA	TGGCTTTTAGGATGGCAAGG
RPL37A	CCAAACGTACCAAGAAAGTCGG	GCGTGCTGGCTGATTTCAA
TSS-TNF	GGGACATATAAAGGCAGTTGTTGG	TCCCTCTTAGCTGGTCCTCTGC
TSS-IL6	AATGAAACCATCCAGCCATCC	CAGAGACGGTGGTCCTCTGC

Supplemental Table 4. GSK761 screen against human BCPs (Bromoscan assay) reveals no binding at K_d of ≤ 30000 nM (for most of tested BCPs) and at K_d of ≤ 21000 nM for PBRM1(5), indicating a high degree of specificity of GSK761 for SP140

Compound Name	DiscoveRx Gene Symbol	K_d (nM)
GSK761	ATAD2A	30000
GSK761	ATAD2B	30000
GSK761	BAZ2A	30000
GSK761	BAZ2B	30000
GSK761	BRD1	30000
GSK761	BRD2(1)	30000
GSK761	BRD2(1,2)	30000
GSK761	BRD2(2)	30000
GSK761	BRD3(1)	30000
GSK761	BRD3(1,2)	30000
GSK761	BRD3(2)	30000
GSK761	BRD4(1)	30000
GSK761	BRD4(1,2)	30000
GSK761	BRD4(2)	30000
GSK761	BRD4(full-length,short-iso.)	30000
GSK761	BRD7	30000
GSK761	BRD8(1)	30000
GSK761	BRD8(2)	30000
GSK761	BRD9	30000
GSK761	BRDT(1)	30000
GSK761	BRDT(1,2)	30000
GSK761	BRDT(2)	30000
GSK761	BRPF1	30000
GSK761	BRPF3	30000
GSK761	CECR2	30000
GSK761	CREBBP	30000
GSK761	EP300	30000
GSK761	FALZ	30000
GSK761	GCN5L2	30000
GSK761	PBRM1(2)	30000
GSK761	PBRM1(5)	21000
GSK761	PCAF	30000
GSK761	SMARCA2	30000
GSK761	SMARCA4	30000
GSK761	TAF1(2)	30000
GSK761	TAF1L(2)	30000
GSK761	TRIM24(Bromo.)	30000
GSK761	TRIM24(PHD,Bromo.)	30000
GSK761	TRIM33(PHD,Bromo.)	30000
GSK761	WDR9(2)	30000
