# Supplementary material for: Modulation of macrophage inflammatory function through selective inhibition of the epigenetic reader protein SP140
Source: BMC Biol. 2022 Aug 19;20:182. doi: 10.1186/s12915-022-01380-6 (PMC9392322; doi:10.1186/s12915-022-01380-6)
Supplement: Supplementary file 16 — Additional file 16. The discovery and the synthesis of GSK761, GSK064, GSK675 and GSK306. [file 12915_2022_1380_MOESM16_ESM.docx]

**Supplementary materials and methods (compound discovery and synthesis)**

**Screening of DNA encoded libraries (GSK761 discovery)**. Affinity screening of DNA Encoded Library (DEL) was done using AntiFlag matrix tips (Phynexus). Before use, the AntiFlag matrix tips were washed four times with 1x selection buffer and stored at 4^°^ C. The SP140 protein (24 kDa) used for the affinity selection was: 6His-Flag-Tev-SP140 (687-867). During the screening process, each AntiFlag matrix tip was loaded with 5 μg of protein and the No target tips was treated with buffer only. The following 1x selection buffer was used in the affinity selection: 50 mM HEPES (7.5), 150 mM NaCl, 0.1% Tween 20, 1 mM BME, 1 mg/mL sheared salmon sperm DNA (sssDNA, Ambion). Three rounds of affinity selection were performed for each screening condition. No target controls (buffer only) were run in parallel as a control.

Selection Round 1: Prior to initiating target selections, 5 μg of SP140 (aa 687-867) protein was immobilized on prepared Anti-Flag resin tip (Phynexus), tips were washed four times with 100 μL of 1 x selection buffer. For each selection, 5 nM of DEL molecules (pool DEL 34-79) in 60 μL of 1 x selection buffer was incubated with the immobilized SP140 (aa 687-867) by pipetting up and down for 1h (RT). Tips were then washed eight times with 100 μL of 1x Selection buffer, and another 2 times with 100 μL of DNA free 1x Selection buffer. In order to release the bound DEL molecules off the tip, a heat elution was performed by treating the tip in 60 μL of 1 x selection buffer (minus sssDNA) for 12 minutes at 80 °C. Eluted samples were post cleared to remove denatured protein by passing over a fresh IMAC resin tip to remove any denatured protein for 10 minutes at RT. This step was repeated once. 1 μL of round 1 elution was retained to be used for qPCR. sssDNA and buffer were added to bring the total volume of the eluted material to 60 uL to be used for next round of selection.

Selection Round 2: The 2^nd^ round selection was performed by binding 5 μg of fresh SP140 (aa 687-867) protein to a fresh prepared Anti-Flag tip. The above selection procedure was repeated using the eluted material from round 1. At the end of round 2, 5 μL of the elution was retained. The eluted material was post cleared twice to remove denatured protein, as described above. sssDNA and buffer were added to bring the volume to 60 μL in order to begin round 3 of the selection.

Selection Round 3: The above selection procedure was repeated with eluted material from round 2. However, no post-clear step was performed for round 3 selection. At the end of round 3 selection, a quantitative PCR was run to assess yield from each round of selection. Target and No target samples from Round 3 elution were sequenced on an Illumina sequencer.

**Cloning, expression and purification**. DNA spanning the SP140 region encoding the Brd and PHD domains (encoding SP140 amino acids 687-867) was prepared in a construct, which was subsequently inserted into a construct that comprised His_6_ and FLAG (DYKDDDDK) affinity chromatography peptides and a TEV protease cleavage site (ENLYFQ\S, “\” denotes the cleaved peptide bond). This DNA sequence was then cloned into a ET11c vector (Bioduro) and then subsequently used to transform *E. coli* BL21 CodonPlus (DE3) RIPL (Stratgene) in media containing 100 μg/mL ampicillin and 34 μg/mL chloramphenicol as selection antibiotics. A culture in Luria Bertani (LB) medium was grown at 30°C, 240 rpm, overnight in Erlenmeyer shake flasks to provide a seed for a larger expression culture in auto-inducible Overnight Express medium (supplemented with 1% Glycerol and 0.1 mM ZnCl_2_), in shake flasks, at 37 °C and 200 rpm. Incubation temperature was reduced to 25°C when O.D.600nm was 1.2. The culture was pelleted by centrifugation after a further 20h incubation and stored at -80° C. For protein purification, a 40 g cell pellet was processed by mixing with 200 mL of PBS, 50 mM Imidazole, 10% Glycerol, PIC III, 1 mg/mL lysozyme, Benzonase (Merck) pH7.4 and left to stir for 30 minutes at 4°C. The resulting suspension was lysed by sonication on ice for 15 minutes (10 seconds on /10 seconds off) and then harvested by centrifugation for 90 minutes at 30000 rpm. The lysate supernatant was collected and then passed through a 20 mL HisTrap HP column (GE Healthcare) and then washed back with buffer A. (Buffer A: PBS, 2 mM DTT, 10% Glycerol, pH 7.4). Bound SP140 was eluted using stepped protocol with buffer B (PBS, 2 mM DTT, 10% Glycerol, 500 mM Imidazole, and pH 7.4) yielding 434.4 mg of total protein. This pool was diluted down to 5 mScm using 20 mM Hepes, 2 mM DTT, 10% Glycerol, and pH 7.5 and further purified using 100 mL Source15Q anion exchange. Elution of the SP140 was carried out using a segmented linear NaCl gradient (0-50% buffer B over 20 column volumes (Cvs), 50-100% over 1 Cv). Three peaks were obtained from the post anion exchange material (Peak 1, 2 and 3) and pooled separately. The pooled fractions from absorbance peak 2 were further purified using gel filtration (Superdex 75 320 mL Cv), which lead to a final yield of purified SP140 protein of 7.87 mg, in a buffer comprising 10 mM Potassium phosphate, 100 mM NaCl, 0.5 mM TCEP, 5% Glycerol , pH7.4.

**Compound synthesis.** Unless otherwise stated, all reactions were carried using anhydrous solvents. Solvents and reagents were purchased from commercial suppliers and used as received. Reactions were monitored by LCMS. Silica flash chromatography was carried out using SP4 apparatus using RediSep® pre-packed silica cartridges. Ion exchange chromatography was carried out using Biotage Isolute cartridges and extracted organic mixtures were dried using Biotage PTFE hydrophobic phase separator frits unless otherwise stated. NMR spectra were recorded at RT (unless otherwise stated) using standard pulse methods on a Bruker AV-400 spectrometer (^1^H = 400 MHz, ^13^C = 101 MHz). Chemical shifts are referenced to trimethylsilane (TMS) or the residual solvent peak, and are reported in ppm. Coupling constants are reported in Hz and refer to ^3^J_H-H_ couplings, unless otherwise stated. Coupling constants are quoted to the nearest 0.1 Hz and multiplicities are given by the following abbreviations and combinations thereof: s (singlet), d (doublet), t (triplet), q (quartet), m (multiplet), br. (broad). LCMS analysis was carried out on a Waters Acquity UPLC instrument equipped with a BEH or CSH column (50 mm x 2.1 mm, 1.7 μm packing diameter) and Waters micromass ZQ MS using alternate-scan positive and negative electrospray. Analytes were detected as a summed UV wavelength of 210 – 350 nm. Two liquid phase methods were used: **Formic**: 40 °C, 1 mL/min flow rate. Gradient elution with the mobile phases as (A) water containing 0.1% volume/volume (v/v) formic acid and (B) acetonitrile containing 0.1% (v/v) formic acid. Gradient conditions were initially 1% B, increasing linearly to 97% B over 1.5 min, remaining at 97% B for 0.4 min then increasing to 100% B over 0.1 min. **High pH**: 40 °C, 1 mL/min flow rate. Gradient elution with the mobile phases as (A) 10 mM aqueous ammonium bicarbonate solution, adjusted to pH 10 with 0.88 M aqueous ammonia and (B) acetonitrile. Gradient conditions were initially 1% B, increasing linearly to 97% B over 1.5 min, remaining at 97% B for 0.4 min then increasing to 100% B over 0.1 min. Mass directed automatic purification (MDAP): **High pH MDAP:** The HPLC analysis was conducted on an Xselect CSH C18 column (150 mm x 30 mm i.d. 5 μm packing diameter) at ambient temperature, eluting with 10 mM ammonium bicarbonate in water adjusted to pH 10 with ammonia solution (solvent A) and acetonitrile (solvent B) using an elution gradient of between 0 and 100% solvent B over 15 or 25 min. The UV detection was an averaged signal from wavelength of 210 nm to 350 nm.  The mass spectra were recorded on a Waters ZQ Mass Spectrometer using alternate-scan positive and negative electrospray. Ionisation data was rounded to the nearest integer.

**GSK761**

**N-(3-(2-(*tert*-Butoxy)ethyl)phenyl)-4-formylbenzamide**

4-Carboxybenzaldehyde (2.3g, 15.32 mmol) was dissolved in Dichloromethane (DCM) (25 mL), then oxalyl chloride (5.83 g, 46.0 mmol) and N,N’-dimethylformamide (DMF) (0.024 mL, 0.306 mmol) were added and the mixture stirred till a colorless solution was obtained. This was evaporated *in vacuo* and the residue redissolved in DCM (25 mL) and cooled in an ice bath. Pyridine (3.72 mL, 46.0 mmol) was added, followed by 3-(2-tertbutoxyethyl)aniline (2.96 g, 15.32 mmol) and the resulting suspension stirred at 0^°^C to room temperature for 1h.

The reaction mixture was diluted with DCM (50 mL), then washed with water (50 mL) and brine (50 mL), dried and evaporated *in vacuo* to a dark brown gum. This was dissolved in DCM (10 mL) and loaded onto a 100 g silica column, then eluted with 0-50% EtOAc/cyclohexane to give *N*-(3-(2-(*tert*-butoxy)ethyl)phenyl)-4-formylbenzamide (2.75 g, 8.45 mmol, 55.2 % yield) as an amber gum.

^1^H NMR (CHLOROFORM-d, 400 MHz) δ 10.13 (s, 1H), 8.0-8.1 (m, 4H), 7.83 (br s, 1H), 7.5-7.6 (m, 2H), 7.33 (t, 1H, *J*=7.6 Hz), 7.0-7.1 (m, 1H), 3.60 (t, 2H, *J*=7.6 Hz), 2.87 (t, 2H, *J*=7.3 Hz), 1.45 (s, 9H) LCMS (2 min Formic): Rt = 1.12 min, [M - H]^+^ = 324

**Methyl 2-(4-((3-(2-(*tert*-butoxy)ethyl)phenyl)carbamoyl)phenyl)-1-methyl-1H-benzo[*d*]imidazole-5-carboxylate**

*N*-(3-(2-(*tert*-butoxy)ethyl)phenyl)-4-formylbenzamide (2.6 g, 7.99 mmol) and methyl 4-(methylamino)-3-nitrobenzoate (1.679 g, 7.99 mmol) were combined in ethanol (50 mL), then sodium dithionite (2.78 g, 15.98 mmol) in water (20 mL) was added and the mixture was stirred at 70^°^C overnight. The mixture was cooled and evaporated *in vacuo* to half volume, then extracted with EtOAc (2 x 50 mL) and the organic layer dried and evaporated *in vacuo* to give a pale yellow solid.

The solid was dissolved in DCM (10 mL) and loaded onto a 100 g silica column, then eluted with 0-80% EtOAc/cyclohexane and product-containing fractions evaporated *in vacuo* to give methyl 2-(4-((3-(2-(*tert*-butoxy)ethyl)phenyl)carbamoyl)phenyl)-1-methyl-1H-benzo[*d*]imidazole-5-carboxylate (2.35 g, 4.84 mmol, 60.6 % yield)

^1^H NMR (CHLOROFORM-d, 400 MHz) δ 8.55 (s, 1H), 8.18 (s, 1H), 8.11 (dd, 1H, *J*=1.0, 8.6 Hz), 8.04 (d, 2H, *J*=8.1 Hz), 7.89 (d, 2H, *J*=8.1 Hz), 7.5-7.6 (m, 2H), 7.46 (d, 1H, *J*=8.6 Hz), 7.33 (t, 1H, *J*=7.6 Hz), 7.09 (d, 1H, *J*=7.6 Hz), 3.98 (s, 3H), 3.93 (s, 3H), 3.60 (t, 2H, *J*=7.6 Hz), 2.87 (t, 2H, *J*=7.3 Hz), 1.20 (s, 9H)

LCMS (2 min Formic): Rt = 1.14 min, [MH]^+^ = 486

**2-(4-((3-(2-(*tert*-Butoxy)ethyl)phenyl)carbamoyl)phenyl)-1-methyl-1H-benzo[*d*]imidazole-5-carboxylic acid**

Methyl 2-(4-((3-(2-(*tert*-butoxy)ethyl)phenyl)carbamoyl)phenyl)-1-methyl-1H-benzo[*d*]imidazole-5-carboxylate (2.3 g, 4.74 mmol) was dissolved in THF (10 mL), then LiOH (0.340 g, 14.21 mmol) in water (5 mL) was added and the solution was heated at 60°Cfor 2h, then cooled to room temperature and evaporated *in vacuo*. The residue was dissolved in water (20 mL) and acidified with 2M HCl to pH 4, then the resulting beige solid collected by filtration and the solid dried in the vacuum oven overnight at 60^°^C to give 2-(4-((3-(2-(*tert*-butoxy)ethyl)phenyl)carbamoyl)phenyl)-1-methyl-1H-benzo[*d*]imidazole-5-carboxylic acid (2.2 g, 4.67 mmol, 98 % yield).

^1^H NMR (DMSO-d_6_, 400 MHz) δ 10.40 (s, 1H), 8.33 (d, 1H, *J*=1.0 Hz), 8.20 (d, 2H, *J*=8.6 Hz), 8.08 (d, 2H, *J*=8.6 Hz), 8.02 (dd, 1H, *J*=1.0, 8.6 Hz), 7.86 (d, 1H, *J*=8.6 Hz), 7.6-7.7 (m, 2H), 7.28 (t, 1H, *J*=7.8 Hz), 7.02 (d, 1H, *J*=7.6 Hz), 4.01 (s, 4H), 3.54 (t, 2H, *J*=7.3 Hz), 2.75 (t, 2H, *J*=7.1 Hz), 1.14 (s, 9H)

LCMS (2 min Formic): Rt = 1.02 min, [MH]^+^ = 472

***N*-(3-(2-(*tert*-Butoxy)ethyl)phenyl)-2-(4-((3-(2-(*tert*-butoxy)ethyl)phenyl)carbamoyl)phenyl)-1-methyl-1H-benzo[*d*]imidazole-5-carboxamide**

To a mixture of 2-(4-((3-(2-(*tert*-butoxy)ethyl)phenyl)carbamoyl)phenyl)-1-methyl-1H-benzo[*d*]imidazole-5-carboxylic acid (100 mg, 0.212 mmol) and 3-(2-(tert-butoxy)ethyl)aniline (62 mg, 0.321 mmol) in DMF (1 mL) were added HATU (121 mg, 0.318 mmol) and DIPEA (0.056 mL, 0.318 mmol) and the reaction mixture was stirred at room temperature for 3 hours. LCMS showed complete consumption of starting material. The solution was then partitioned between EtOAc and water. The organic layer was washed with water (3x 10 mL), dried over magnesium sulphate and evaporated under vacuum. The sample was loaded in DCM and purified on a 10 g silica cartridge eluting with 0-100% EtOAc-cyclohexane. The appropriate fractions were combined and evaporated *in vacuo* to give the required product *N*-(3-(2-(*tert*-butoxy)ethyl)phenyl)-2-(4-((3-(2-(*tert*-butoxy)ethyl)phenyl)carbamoyl)phenyl)-1-methyl-1H-benzo[d]imidazole-5-carboxamide (118 mg, 0.182 mmol, 86 % yield), as a colorless glass.

^1^HNMR (CHLOROFORM-d ,400MHz): δ (ppm) 8.28 (d, *J*=1.3 Hz, 1H), 8.01 - 8.06 (m, 3H), 7.94 - 7.99 (m, 2H), 7.89 (d, *J*=8.3 Hz, 2H), 7.56 (s, 2H), 7.52 - 7.56 (m, 2H), 7.50 (d, *J*=8.6 Hz, 1H), 7.27 - 7.36 (m, 2H), 7.01 - 7.10 (m, 2H), 3.94 (s, 3H), 3.58 (t, *J*=7.5 Hz, 4H), 2.86 (t, *J*=7.5 Hz, 4H), 1.19 (s, 18H)

LCMS (2 min Formic): Rt = 1.30 min, [MH]^+^ = 647

**GSK064**

**4-((2,2-Dimethyl-4-oxo-3,8,11-trioxa-5-azatridecan-13-yl)amino)-3-nitrobenzoic acid**

*N*-Boc-2,2'-(ethylenedioxy)diethylamine (1.300 mL, 5.48 mmol) and diisopropylethylamine (2.781 mL, 16.25 mmol) were added to a stirred solution of 4-fluoro-3-nitrobenzoic acid (1.0033 g, 5.42 mmol) in ethanol (15 mL) at ambient temperature. The resulting mixture was stirred under N_2_ atmosphere at 80 °C for 3.5 hr. The solvent was evaporated and dried *in vacuo* to give the crude product, 4-((2,2-dimethyl-4-oxo-3,8,11-trioxa-5-azatridecan-13-yl)amino)-3-nitrobenzoic acid (3.5849 g, 8.67 mmol, 160 % yield) as an orange/yellow oil.

The crude product was used in the next step without further purification.

^1^H NMR (CHLOROFORM-d, 400 MHz) δ 8.92 (d, 1H, *J*=2.0 Hz), 8.43 (br t, 1H, *J*=4.8 Hz), 8.16 (dd, 1H, *J*=1.8, 8.8 Hz), 6.84 (d, 1H, *J*=8.6 Hz), 5.1 (br s, 1H), 3.81 (t, 2H, *J*=5.3 Hz), 3.6-3.7 (m, 4H), 3.5-3.6 (m, 4H), 3.3-3.4 (m, 2H), 1.40 (s, 9H)

LCMS (2 min Formic): Rt = 0.92 min, [MH+]= 414.

**3-Amino-4-((2,2-dimethyl-4-oxo-3,8,11-trioxa-5-azatridecan-13-yl)amino)benzoic acid**

Under N_2_ atmosphere ethanol (10 mL) was to added palladium, 10 wt. % (dry basis) on activated carbon, wet, Degussa type E101 NE/W (0.3776 g, 3.55 mmol) in a hydrogenation flask. A solution of the crude starting material 4-((2,2-dimethyl-4-oxo-3,8,11-trioxa-5-azatridecan-13-yl)amino)-3-nitrobenzoic acid (3.548 g equivalent to 2.241 g, 5.42 mmol) dissolved in ethanol (15 mL) was then added to the catalyst mixture under vacuum. The resulting mixture was placed under an atmosphere of hydrogen gas at room temperature and pressure and stirred vigorously. Once hydrogen uptake had ceased, the reaction mixture was filtered through Celite™ under nitrogen. The solvent was evaporated from the filtrate *in vacuo* to leave a residue that was dried to give the crude product, 3-amino-4-((2,2-dimethyl-4-oxo-3,8,11-trioxa-5-azatridecan-13-yl)amino)benzoic acid (3.141 g, 8.19 mmol, 151 % yield), as a brown oil, which was used without purification in the next step.

LCMS (2 min Formic): Rt = 0.73 min, [MH+]= 384.

**2-(4-Carboxyphenyl)-1-(2,2-dimethyl-4-oxo-3,8,11-trioxa-5-azatridecan-13-yl)-1H-benzo[I]imidazole-5-carboxylic acid**

Acetic acid (14 mL) was added to the crude 3-amino-4-((2,2-dimethyl-4-oxo-3,8,11-trioxa-5-azatridecan-13-yl)amino)benzoic acid (2.350 g crude, equivalent to 1.557 g, 4.06 mmol) and 4-carboxybenzaldehyde (0.6122 g, 4.08 mmol). The resulting mixture was stirred under nitrogen at room temperature for 66 hr. The solvent was evaporated *in vacuo* to give a dark reddish-brown oil. The oil was loaded onto a 100 g silica cartridge as a partial suspension in dichloromethane and then purified by silica flash chromatography, eluting with 0-100 % of (1:10:89 acetic acid:MeOH:DCM)/DCM. The required fractions were combined and the solvent evaporated *in vacuo* to give the final product, 2-(4-carboxyphenyl)-1-(2,2-dimethyl-4-oxo-3,8,11-trioxa-5-azatridecan-13-yl)-1H-benzo[I]imidazole-5-carboxylic acid (799.2 mg, 1.323 mmol, 32.6 % yield), as a brown crunchy foam.

^1^H NMR (400 MHz, METHANOL-d_4_) δ 8.62 (s, 1H), 8.43 – 8.37 (m, 2H), 8.26 (dd, *J* = 8.6, 1.5 Hz, 1H), 8.18 – 8.14 (m, 2H), 7.96 – 7.92 (m, 1H), 4.76 – 4.71 (m, 2H), 4.07 – 4.02 (m, 2H), 3.70 – 3.56 (m, 2H), 3.53 – 3.46 (m, 4H), 3.28 – 3.25 (m, 2H), 1.59 (s, 9H)

LCMS (2 min Formic): Rt = 0.78 min, [MH+]= 514

***tert*-Butyl (2-(2-(2-(5-((3-(2-(tert-butoxy)ethyl)phenyl)carbamoyl)-2-(4-((3-(2-(*tert*-butoxy)ethyl)phenyl)carbamoyl)phenyl)-1H-benzo[*d*]imidazol-1-yl)ethoxy)ethoxy)ethyl)carbamate**

Diisopropylethylamine (0.400 mL, 2.337 mmol) was added to a solution of 3-(2-(*tert*-butoxy)ethyl)aniline (181.3 mg, 0.938 mmol), 2-(4-carboxyphenyl)-1-(2,2-dimethyl-4-oxo-3,8,11-trioxa-5-azatridecan-13-yl)-1H-benzo[*d*]imidazole-5-carboxylic acid (399.4 mg, 0.778 mmol) and HATU (356.7 mg, 0.938 mmol) in DMF (5 mL) and the resulting mixture stirred at room temperature under nitrogen for 150 min. The solvent was evaporated under a stream of nitrogen, citric acid (10 % aq) (50 mL) was added and the aqueous phase extracted with EtOAc (3 x 50 mL). The combined organic phases were filtered through a hydrophobic frit and the solvent evaporated *in vacuo* to give a brown residue. The residue was dissolved in MeOH and loaded onto a 50 g silica cartridge, which was then dried *in vacuo* and eluted with 30-100 % (1 % acetic acid in ethyl acetate)/cyclohexane. The desired product and mono-coupled by-products co-eluted and fractions containing the desired products were combined and the solvent evaporated *in vacuo* to give a pale yellow residue.

The resulting residue was dissolved in dichloromethane and loaded on to a 50g silica cartridge, then eluted with 0-10 % MeOH/DCM to give *tert*-butyl (2-(2-(2-(5-((3-(2-(*tert*-butoxy)ethyl)phenyl)carbamoyl)-2-(4-((3-(2-(tert-butoxy)ethyl)phenyl)carbamoyl)phenyl)-1H-benzo[*d*]imidazol-1-yl)ethoxy)ethoxy)ethyl)carbamate (125.7 mg, 0.131 mmol, 16.83 % yield as a pale yellow solid.

^1^H NMR (CHLOROFORM-d, 400 MHz) δ 8.3-8.4 (m, 1H), 7.9-8.2 (m, 6H), 7.6-7.7 (m, 5H), 7.3-7.4 (m, 2H), 7.07 (t, 2H, *J*=7.1 Hz), 4.8-5.0 (m, 1H), 4.4-4.6 (m, 2H), 3.92 (br s, 2H), 3.60 (t, 4H, *J*=7.6 Hz), 3.49 (br s, 4H), 3.3-3.4 (m, 2H), 3.1-3.2 (m, 2H), 2.8-2.9 (m, 4H), 1.43 (s, 9H), 1.21 (s, 18H)

LCMS (2 min Formic): Rt = 1.40 min, [MH+] = 864 .

**1-(2-(2-(2-Aminoethoxy)ethoxy)ethyl)-*N*-(3-(2-(*tert*-butoxy)ethyl)phenyl)-2-(4-((3-(2-(*tert*-butoxy)ethyl)phenyl)carbamoyl)phenyl)-1H-benzo[*d*]imidazole-5-carboxamide**

Hydrogen chloride solution (4.0 M in dioxane, 2 mL, 8.00 mmol) was added to *tert*-butyl (2-(2-(2-(5-((3-(2-(*tert*-butoxy)ethyl)phenyl)carbamoyl)-2-(4-((3-(2-(*tert*-butoxy)ethyl)phenyl)carbamoyl)phenyl)-1H-benzo[*d*]imidazol-1-yl)ethoxy)ethoxy)ethyl)carbamate (112.1 mg, 0.130 mmol) and the resulting mixture stirred for 2 min at room temperature. The reaction was quenched with saturated sodium carbonate solution (2 mL), then water (3 mL) and EtOAc (5 mL) were added and the phases separated. The aqueous phase was extracted with further ethyl acetate (2 x 5 mL) and the combined organic phases were filtered through a hydrophobic frit and the solvent evaporated. The resulting residue was dissolved in methanol (1 mL) and purified by high pH MDAP to give

1-(2-(2-(2-aminoethoxy)ethoxy)ethyl)-N-(3-(2-(*tert*-butoxy)ethyl)phenyl)-2-(4-((3-(2-(*tert*-butoxy)ethyl)phenyl)carbamoyl)phenyl)-1H-benzo[*d*]imidazole-5-carboxamide (46.1 mg, 0.057 mmol, 44.2 % yield), was obtained as a very pale yellow glass.

^1^H NMR (CHLOROFORM-d, 400 MHz) δ 8.72 (s, 1H), 8.3-8.4 (m, 2H), 8.0-8.1 (m, 4H), 7.95 (dd, 1H, *J*=1.8, 8.3 Hz), 7.5-7.6 (m, 4H), 7.32 (dt, 2H, *J*=3.5, 7.8 Hz), 7.07 (t, 2H, *J*=7.8 Hz), 4.51 (t, 2H, *J*=5.3 Hz), 3.91 (t, 3H, *J*=5.1 Hz), 3.60 (t, 6H, *J*=7.3 Hz), 3.5-3.6 (m, 4H), 3.39 (t, 2H, *J*=5.3 Hz), 2.88 (t, 4H, *J*=7.6 Hz), 2.80 (t, 2H, *J*=5.3 Hz), 1.21 (s, 18H)

LCMS (2 min high pH): Rt = 1.27 min, [MH+]= 764

**3-(2-((1E,3E,5Z)-5-(3-(6-((2-(2-(2-(5-((3-(2-(*tert*-Butoxy)ethyl)phenyl)carbamoyl)-2-(4-((3-(2(*tert*-butoxy)ethyl)phenyl)carbamoyl)phenyl)-1H-benzo[*d*]imidazol-1yl)ethoxy)ethoxy)ethyl)amino)6-oxohexyl)-3-methyl-5-sulfo-1-(3-sulfopropyl)indolin-2-ylidene)penta-1,3-dien-1-yl)-3,3-dimethyl-5-sulfo-3H-indol-1-ium-1-yl)propane-1-sulfonate, 3 Ammonia salt**

Alexa Fluor 647 carboxylic acid succinimidyl ester (5mg) (Invitrogen) on 15th July 2011; Lot 890799; cat# A20106; mf not given; Exact Mass: not given; Molecular Weight: not given but cited as "~1250".

Structure of AlexaFluor 647 determined from WO02/26891 Compound 9. This structure contains two pendant propyl sulfonic acids contrary to some reports suggesting that the molecule contains butyl sulfonic acids. Salt form from patent example of tris(triethylamine) has MWt. 1259.67 which approximates to the vendor's citation of "~1250".

3-(2-((1E,3E,5Z)-5-(3-(6-((2,5-dioxopyrrolidin-1-yl)oxy)-6-oxohexyl)-3-methyl-5-sulfo-1-(3-sulfopropyl)indolin-2-ylidene)penta-1,3-dien-1-yl)-3,3-dimethyl-5-sulfo-3H-indol-1-ium-1-yl)propane-1-sulfonate, 3 N,N-diethylethanamine salt (5 mg, 3.97 µmol) in anhydrous DMF (200 µL) was added to 1-(2-(2-(2-aminoethoxy)ethoxy)ethyl)-N-(3-(2-(tert-butoxy)ethyl)phenyl)-2-(4-((3-(2-(tert-butoxy)ethyl)phenyl)carbamoyl)phenyl)-1H-benzo[d]imidazole-5-carboxamide (5.4 mg, 7.07 µmol). Diisopropylethylamine (1.4 µL, 8.02 µmol) was added and the resulting mixture stirred at room temperature in a vial wrapped in foil for 16 hr. Acetonitrile was added to increase the sample volume to 1 mL. The sample was then purified by high pH MDAP to give 3-(2-((1E,3E,5Z)-5-(3-(6-((2-(2-(2-(5-((3-(2-(*tert*-butoxy)ethyl)phenyl)carbamoyl)-2-(4-((3-(2-(*tert*-butoxy)ethyl)phenyl)carbamoyl)phenyl)-1H-benzo[*d*]imidazol-1-yl)ethoxy)ethoxy)ethyl)amino)-6-oxohexyl)-3-methyl-5-sulfo-1-(3-sulfopropyl)indolin-2-ylidene)penta-1,3-dien-1-yl)-3,3-dimethyl-5-sulfo-3H-indol-1-ium-1-yl)propane-1-sulfonate, 3 Ammonia salt (6.7 mg, 3.84 µmol, 97 % yield), as a blue solid.

^1^H NMR (METHANOL-d_4_, 400 MHz) δ 8.2-8.5 (m, 3H), 8.17 (d, 2H, *J*=8.6 Hz), 8.0-8.1 (m, 3H), 7.9-7.9 (m, 3H), 7.8-7.9 (m, 2H), 7.6-7.7 (m, 4H), 7.43 (dd, 2H, *J*=8.3, 10.9 Hz), 7.31 (t, 2H, *J*=7.8 Hz), 7.07 (dd, 2H, *J*=3.8, 7.3 Hz), 6.6-6.8 (m, 1H), 6.45 (br dd, 2H, *J*=5.3, 13.4 Hz), 4.57 (t, 2H, *J*=4.8 Hz), 4.3-4.4 (m, 4H), 3.88 (t, 2H, *J*=5.1 Hz), 3.65 (dt, 6H, *J*=2.5, 7.1 Hz), 3.42 (br d, 6H, *J*=3.5 Hz), 3.1-3.2 (m, 2H), 2.9-3.1 (m, 5H), 2.8-2.9 (m, 5H), 2.2-2.3 (m, 5H), 1.92 (s, 2H), 1.73 (s, 4H), 1.70 (s, 7H), 1.4-1.5 (m, 2H), 1.17 (s, 18H), 1.1-1.2 (m, 1H), 0.83 (br d, 1H, *J*=3.0 Hz), 0.5-0.7 (m, 1H)

LCMS (2 min high pH): Rt = 0.88 min, [M+2H]2+= 804

**GSK675**

***N*-(3-(2-(tert-Butoxy)ethyl)phenyl)-4-chloro-3-nitrobenzamide**

4-Chloro-3-nitrobenzoic acid (4194.8 mg, 20.81 mmol) was taken up into DCM (100 mL) in an ice-bath under nitrogen. Oxalyl dichloride (5.45 mL, 62.4 mmol) was slowly added to the mixture followed by DMF (2 drops), at which point the mixture started to slightly bubble. The reaction was then allowed to stir at RT for 2.5h then the volatiles removed *in vacuo*.

The crude acid chloride was taken up into DCM (100 mL) in an ice-bath under nitrogen. 3-(2-(*tert*-butoxy)ethyl)aniline (4023 mg, 20.81 mmol) was slowly added followed by pyridine (3.36 mL, 41.6 mmol). The reaction was allowed to stir at RT for 2h, then evaporated *in vacuo* to give a brown thick oil. The crude was dissolved in DCM (40 mL) and washed with saturated aq sodium bicarbonate solution (2 x 40 mL) then with hydrochloric acid (1M aq, 40 mL), dried with magnesium sulfate and filtered under vacuum. The solvent was evaporated under vacuum to give *N*-(3-(2-(*tert*-butoxy)ethyl)phenyl)-4-chloro-3-nitrobenzamide (8132.3 mg, 20.50 mmol, 99 % yield) as a brown oil.

^1^H NMR (DMSO-d_6_, 400 MHz) δ 10.46 (s, 1H), 8.64 (d, 1H, *J*=2.3 Hz), 8.27 (dd, 1H, *J*=2.1, 8.5 Hz), 7.97 (d, 1H, *J*=8.6 Hz), 7.6-7.7 (m, 2H), 7.2-7.4 (m, 1H), 7.03 (d, 1H, *J*=7.6 Hz), 3.52 (t, 2H, *J*=7.1 Hz), 2.74 (t, 2H, *J*=7.1 Hz), 1.12 (s, 9H)

LCMS (2 min Formic): Rt = 1.28 min, [M - H]^+^ = 375, 377 (1 Cl)

**4-((2-((2-Aminoethyl)(methyl)amino)ethyl)amino)-N-(3-(2-(tert-butoxy)ethyl)phenyl)-3-nitrobenzamide**

N-(3-(2-(*tert*-Butoxy)ethyl)phenyl)-4-chloro-3-nitrobenzamide (1.03g, 2.73 mmol) and N1-(2-aminoethyl)-*N*1-methylethane-1,2-diamine (0.480 g, 4.10 mmol) were stirred in DMF (10 mL) and triethylamine (0.381 mL, 2.73 mmol) for 3h at 50^°^C, then the solution was cooled and diluted with water (50ml), extracted with EtOAc (2 x 50 mL) and the organic layer washed with water (2 x 50 mL), dried and evaporated *in vacuo*. The residue was loaded onto a 50 g silica column and eluted with 0-20% 2 M methanolic ammonia/DCM to give 4-((2-((2-aminoethyl)(methyl)amino)ethyl)amino)-*N*-(3-(2-(*tert*-butoxy)ethyl)phenyl)-3-nitrobenzamide (0.85g, 1.858 mmol, 68.0 % yield) as a bright yellow gum

^1^H NMR (CHLOROFORM-d, 400 MHz) δ 8.7-8.8 (m, 1H), 8.67 (d, 1H, *J*=2.5 Hz), 8.52 (s, 1H), 8.0-8.1 (m, 1H), 7.5-7.6 (m, 2H), 7.2-7.3 (m, 1H), 7.00 (d, 1H, *J*=7.6 Hz), 6.85 (d, 1H, *J*=9.1 Hz), 3.56 (t, 2H, *J*=7.3 Hz), 3.38 (q, 2H, *J*=5.6 Hz), 2.8-2.9 (m, 4H), 2.7-2.8 (m, 2H), 2.4-2.6 (m, 2H), 2.28 (s, 3H), 1.17 (s, 9H)

LCMS (2 min high pH): Rt = 1.10 min, [MH]^+^ = 458

***tert*-Butyl (2-((2-((4-((3-(2-(tert-butoxy)ethyl)phenyl)carbamoyl)-2-nitrophenyl)amino)ethyl)(methyl)amino)ethyl)carbamate**

4-((2-((2-Aminoethyl)(methyl)amino)ethyl)amino)-*N*-(3-(2-(tert-butoxy)ethyl)phenyl)-3-nitrobenzamide (0.84g, 1.836 mmol) and Boc_2_O (0.426 mL, 1.836 mmol) were dissolved in DCM (20 mL) and allowed to stand for 2h, then the solvent was evaporated *in vacuo* and the residue was purified by chromatography on a 50 g silica column eluting with 0-10% 2 M methanolic ammonia/DCM to give *tert*-butyl (2-((2-((4-((3-(2-(tert-butoxy)ethyl)phenyl)carbamoyl)-2-nitrophenyl)amino)ethyl)(methyl)amino)ethyl)carbamate (0.85g, 1.524 mmol, 83 % yield) as a bright yellow gum.

^1^H NMR (CHLOROFORM-d, 400 MHz) δ 8.7-8.9 (m, 1H), 8.69 (br s, 1H), 7.9-8.2 (m, 2H), 7.5-7.6 (m, 2H), 7.2-7.4 (m, 1H), 7.03 (d, 1H, *J*=7.6 Hz), 6.90 (d, 1H, *J*=9.1 Hz), 5.10 (br s, 1H), 3.58 (t, 2H, *J*=7.3 Hz), 3.40 (q, 2H, *J*=5.6 Hz), 3.27 (q, 2H, *J*=5.6 Hz), 2.85 (t, 2H, *J*=7.6 Hz), 2.77 (t, 2H, *J*=5.8 Hz), 2.58 (t, 2H, *J*=5.8 Hz), 2.34 (s, 3H), 1.40 (s, 9H), 1.19 (s, 9H)

LCMS (2 min high pH): Rt = 1.41 min, [MH]^+^ = 558

**1-(2-((2-Aminoethyl)(methyl)amino)ethyl)-*N*-(3-(2-(*tert*-butoxy)ethyl)phenyl)-2-(4-((3-(2-(*tert*-butoxy)ethyl)phenyl)carbamoyl)phenyl)-1H-benzo[*d*]imidazole-5-carboxamide**

*tert*-Butyl (2-((2-((4-((3-(2-(*tert*-butoxy)ethyl)phenyl)carbamoyl)-2-nitrophenyl)amino)ethyl)(methyl)amino)ethyl)carbamate (0.21g, 0.377 mmol) and *N*-(3-(2-(*tert*-butoxy)ethyl)phenyl)-4-formylbenzamide (0.123 g, 0.377 mmol) were dissolved in ethanol (10 mL), then a solution of sodium dithionite (0.197 g, 1.130 mmol) in water (3 mL) was added and the mixture was heated at reflux for 3h. The solvent was evaporated *in vacuo* and the residue was partitioned between EtOAc (10 mL) and water (5 mL), the organic layer dried and evaporated to give a pale yellow gum.

The crude product was dissolved in DCM (3 mL) and HCl (1M in ether, 1 mL, 1.000 mmol) was added, the mixture stirred for 2h, then evaporated *in vacuo*. A sample (20mg) of the crude product was purified by high pH MDAP to give 1-(2-((2-aminoethyl)(methyl)amino)ethyl)-N-(3-(2-(*tert*-butoxy)ethyl)phenyl)-2-(4-((3-(2-(*tert*-butoxy)ethyl)phenyl)carbamoyl)phenyl)-1H-benzo[*d*]imidazole-5-carboxamide (10mg, 0.014 mmol, 3.62 % yield) as a pale yellow gum.

The remaining crude amine was purified by chromatography on silica (25 g column) eluting with 0-20% 2 M methanolic ammonia/DCM to give 1-(2-((2-aminoethyl)(methyl)amino)ethyl)-N-(3-(2-(*tert*-butoxy)ethyl)phenyl)-2-(4-((3-(2-(*tert*-butoxy)ethyl)phenyl)carbamoyl)phenyl)-1H-benzo[*d*]imidazole-5-carboxamide (85 mg, 0.116 mmol, 30.8 % yield) as a colorless foam.

^1^H NMR (CHLOROFORM-d, 400 MHz) δ 8.48 (s, 1H), 8.27 (s, 1H), 8.14 (s, 1H), 8.03 (d, 3H, *J*=8.1 Hz), 7.96 (dd, 1H, *J*=1.5, 8.6 Hz), 7.87 (d, 3H, *J*=8.6 Hz), 7.5-7.6 (m, 7H), 7.3-7.4 (m, 3H), 7.07 (t, 2H, *J*=8.8 Hz), 4.40 (br t, 2H, *J*=6.6 Hz), 3.60 (dt, 5H, *J*=1.8, 7.5 Hz), 2.87 (t, 5H, *J*=7.3 Hz), 2.71 (t, 2H, *J*=6.3 Hz), 2.4-2.5 (m, 2H), 2.3-2.4 (m, 2H), 2.09 (s, 3H), 1.20 (s, 18H)

LCMS (2 min high pH): Rt = 1.27 min, [MH]^+^ = 733

***N*-(3-(2-(*tert*-Butoxy)ethyl)phenyl)-2-(4-((3-(2-(*tert*-butoxy)ethyl)phenyl)carbamoyl)phenyl)-1-(2-(methyl(2-(6-(5-((3aS,4S,6aR)-2-oxohexahydro-1H-thieno[3,4-d]imidazol-4-yl)pentanamido)hexanamido)ethyl)amino)ethyl)-1H-benzo[*d*]imid**

**azole-5-carboxamide**

1-(2-((2-Aminoethyl)(methyl)amino)ethyl)-N-(3-(2-(*tert*-butoxy)ethyl)phenyl)-2-(4-((3-(2-(*tert*-butoxy)ethyl)phenyl)carbamoyl)phenyl)-1H-benzo[*d*]imidazole-5-carboxamide (15 mg, 0.020 mmol) and 2,5-dioxopyrrolidin-1-yl 6-(5-((3aS,4S,6aR)-2-oxohexahydro-1H-thieno[3,4-d]imidazol-4-yl)pentanamido)hexanoate (9.30 mg, 0.020 mmol) were dissolved in DMF (0.9 mL) and triethylamine (8.56 µl, 0.061 mmol) and the solution was allowed to stand overnight. LCMS (2 min high pH ): Rt = 1.21 min, [MH]^+^ = 1072. The solution was purified by high pH MDAP to give *N*-(3-(2-(*tert*-butoxy)ethyl)phenyl)-2-(4-((3-(2-(*tert*-butoxy)ethyl)phenyl)carbamoyl)phenyl)-1-(2-(methyl(2-(6-(5-((3aS,4S,6aR)-2-oxohexahydro-1H-thieno[3,4-d]imidazol-4-yl)pentanamido)hexanamido)ethyl)amino)ethyl)-1H-benzo[*d*]imidazole-5-carboxamide (8.5 mg, 7.93 µmol, 38.7 % yield) as a colorless solid.

^1^H NMR (METHANOL-d_4_, 400 MHz) δ 8.39 (d, 1H, *J*=1.5 Hz), 8.19 (d, 2H, *J*=8.6 Hz), 8.04 (dd, 1H, *J*=1.5, 8.6 Hz), 8.0-8.0 (m, 2H), 7.83 (d, 1H, *J*=8.6 Hz), 7.5-7.7 (m, 4H), 7.32 (dt, 2H, *J*=3.0, 7.8 Hz), 7.08 (t, 2H, *J*=6.8 Hz), 4.54 (t, 2H, *J*=6.1 Hz), 4.47 (dd, 1H, *J*=5.1, 8.1 Hz), 4.2-4.3 (m, 1H), 3.66 (t, 5H, *J*=7.3 Hz), 3.1-3.2 (m, 1H), 3.00 (t, 2H, *J*=6.6 Hz), 2.8-2.9 (m, 5H), 2.8-2.8 (m, 2H), 2.6-2.7 (m, 1H), 2.37 (t, 2H, *J*=6.3 Hz), 2.17 (t, 2H, *J*=7.3 Hz), 2.06 (t, 2H, *J*=7.6 Hz), 1.4-1.8 (m, 11H), 1.3-1.3 (m, 3H), 1.21 (s, 18H)

LCMS (2 min high pH ): Rt = 1.04 min, M/2+H observed 536

**GSK306**

***N*-(3-(2-(*tert*-Butoxy)ethyl)phenyl)-2-(4-((3-(2-(*tert*-butoxy)ethyl)phenyl)carbamoyl)phenyl)-1-(2-(2-(2-(3',6'-dihydroxy-3-oxo-3H-spiro[isobenzofuran-1,9'-xanthen]-5-ylcarboxamido)ethoxy)ethoxy)ethyl)-1H-benzo[*d*]imidazole-5-carboxamide**

DMF (0.4 mL) and triethylamine (4.42 µL, 0.032 mmol) were added to 5-carboxyfluorescein N-succinimidyl ester (5.1 mg, 10.77 µmol) and 1-(2-(2-(2-aminoethoxy)ethoxy)ethyl)-N-(3-(2-(tert-butoxy)ethyl)phenyl)-2-(4-((3-(2-(tert-butoxy)ethyl)phenyl)carbamoyl)phenyl)-1H-benzo[d]imidazole-5-carboxamide (13.5 mg, 0.018 mmol) and the resulting mixture stirred at room temperature in a vial wrapped in foil for 17.5 hr. The mixture was left to stand overnight, then diluted with methanol to 1 mL and then the sample purified by MDAP (high pH) to give *N*-(3-(2-(*tert*-butoxy)ethyl)phenyl)-2-(4-((3-(2-(tert-butoxy)ethyl)phenyl)carbamoyl)phenyl)-1-(2-(2-(2-(3',6'-dihydroxy-3-oxo-3H-spiro[isobenzofuran-1,9'-xanthen]-5-ylcarboxamido)ethoxy)ethoxy)ethyl)-1H-benzo[*d*]imidazole-5-carboxamide (6.8 mg, 5.76 µmol, 53.4 % yield), as an orange solid.

^1^H NMR (METHANOL-d_4_, 400 MHz) δ 8.37 (dd, 2H, *J*=1.3, 11.9 Hz), 8.1-8.2 (m, 2H), 8.0-8.1 (m, 3H), 7.98 (dd, 1H, *J*=1.5, 8.6 Hz), 7.80 (d, 1H, *J*=8.6 Hz), 7.6-7.7 (m, 2H), 7.58 (br d, 2H, *J*=7.6 Hz), 7.2-7.3 (m, 2H), 7.21 (d, 1H, *J*=8.1 Hz), 7.0-7.1 (m, 2H), 6.6-6.8 (m, 4H), 6.5-6.6 (m, 2H), 4.57 (br t, 2H, *J*=4.8 Hz), 3.94 (br t, 2H, *J*=5.1 Hz), 3.63 (dt, 4H, *J*=2.0, 7.1 Hz), 3.5-3.6 (m, 8H), 2.81 (br t, 4H, *J*=6.6 Hz), 1.18 (m, 18H) (5 exchangeable protons not seen).

LCMS (2 min high pH ): Rt = 0.98 min, [MH+]= 1122
